# Supplementary material for: Identification and characterization of constrained non-exonic bases lacking predictive epigenomic and transcription factor binding annotations
Source: Nat Commun. 2020 Dec 2;11:6168. doi: 10.1038/s41467-020-19962-9 (PMC7710766; doi:10.1038/s41467-020-19962-9)
Supplement: Supplementary file 1 — Supplementary Information [file 41467_2020_19962_MOESM1_ESM.pdf]

Supplementary Information

Identification and characterization of constrained non-exonic bases lacking predictive epigenomic and transcription factor binding annotations

Grujic et al.

Contents:

Supplementary Figures 1-28

Supplementary Tables 1-3

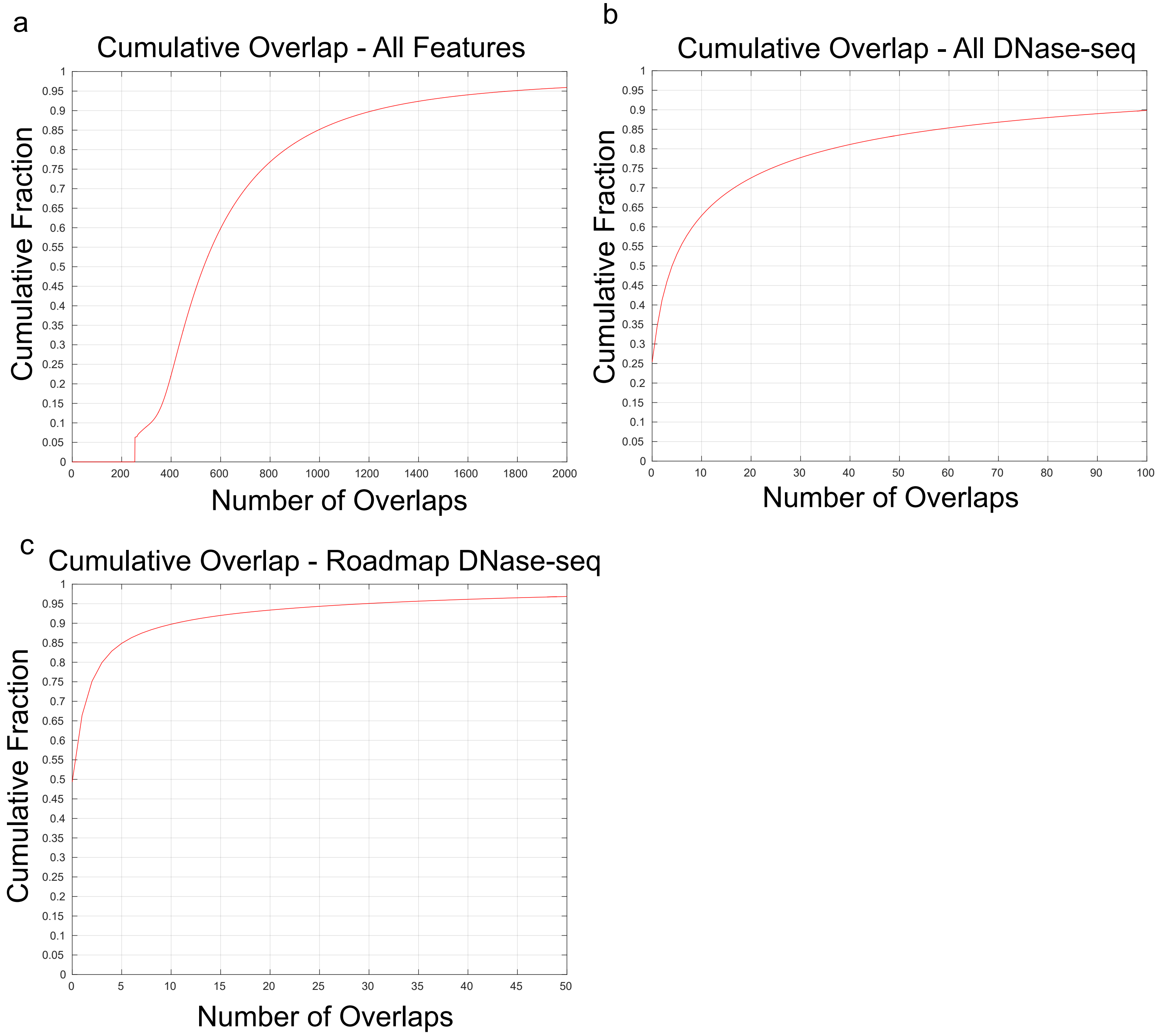

Supplementary Figure 1: Cumulative Distribution of Overlap with Features. (a) The figure shows the cumulative distribution of the number of features of the 63,741 that overlap a base in the genome. **(b)** The same as (a) except restricted to the subset of 4,522 features corresponding to a DNase-seq experiment. **(c)** The same as (b) but further restricted to the subset of 350 DNase-seq experiments uniformly processed by the Roadmap Epigenomics Consortium. This shows that the majority of bases in the genome are overlapped by one or more of these features. Source data are provided as a Source Data file.

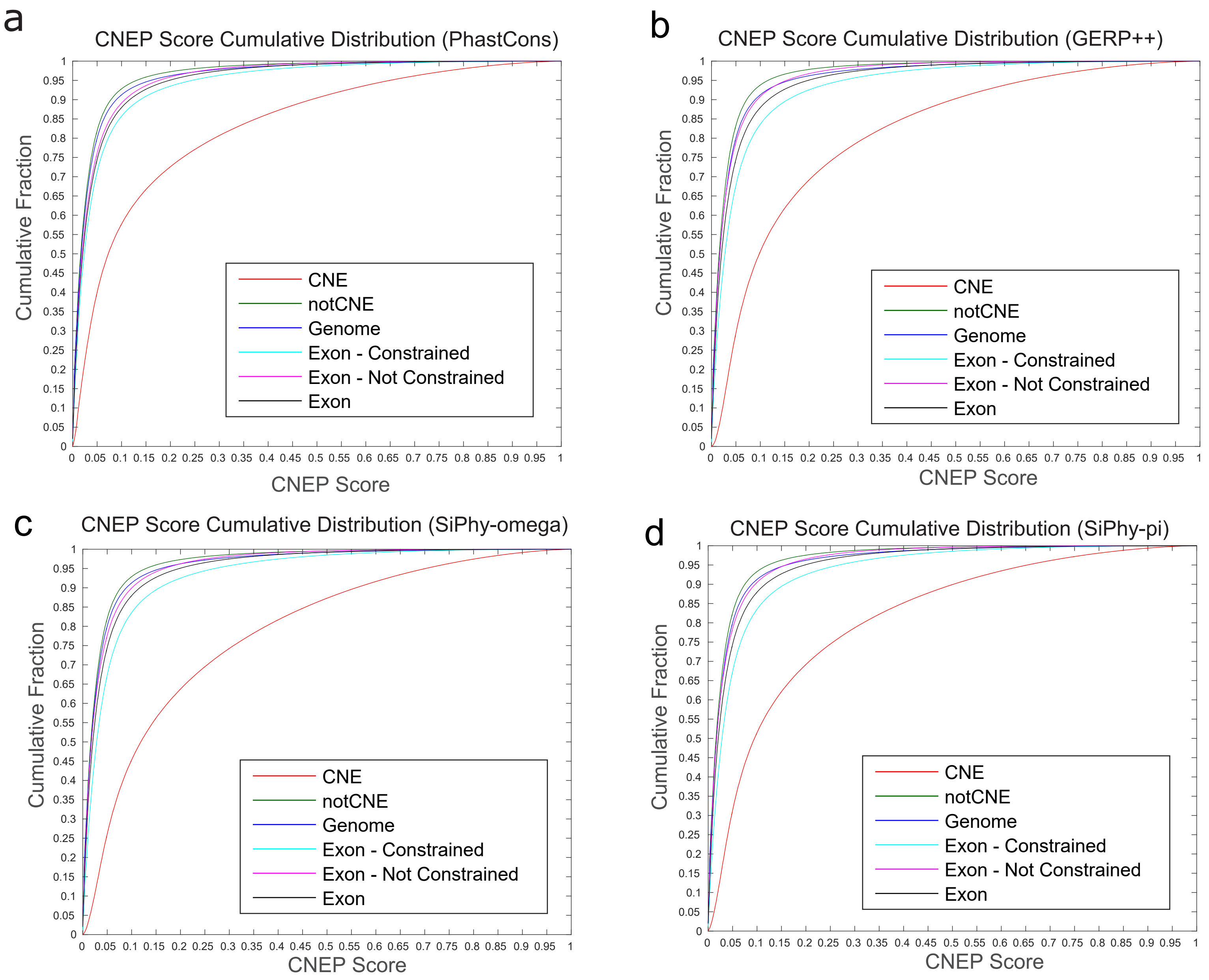

e

Genome Coverage %

set	GERP++	PhastCons	SiPhy-omega	SiPhy-pi
High_CNE	4.1	2.5	2.4	3.2
Low_CNE	1.3	1.3	0.6	1.1
CNE	5.3	3.9	3.0	4.4
High_notCNE	19.6	21.1	21.3	20.4
Low_notCNE	71.1	71.0	71.7	71.2
notCNE	90.7	92.1	93.0	91.6

f

Non-exonic bases

	CNEP below average (<= 0.0419)	CNEP above average (> 0.0419)
CNE	Low_CNE	High_CNE
notCNE	Low_notCNE	High_notCNE

Supplementary Figure 2: Cumulative distribution of CNEP scores and genome coverage. (a) Similar plot to **Fig. 2a**, the graph shows the cumulative distribution of the CNEP score genome-wide, in constrained non-exonic (CNE) bases, and bases that are not in constrained elements and also not in exons (notCNE) for PhastCons. This plot is extended to also show the cumulative distribution of the CNEP score within exons (Exon), bases within exons that overlap a constrained element (Exon – Constrained), and bases within exons that do not overlap a constrained element (Exon – Not Constrained). Similar plots to (a), but showing plots for (b) GERP++, (c) SiPhy-omega and (d) SiPhy-pi constrained element sets. (e) The percent of genome covered by High_CNE, Low_CNE, CNE, High_notCNE, Low_notCNE, and notCNE bases. (f) Schematic illustration of the relationship between High_CNE, Low_CNE, CNE, High_notCNE, Low_notCNE, notCNE bases, and the CNEP score. The High_CNE, Low_CNE, High_notCNE, and Low_notCNE only contain non-exonic bases and all non-exonic bases are in one of those four sets. Source data are provided as a Source Data file.

Constrained element coverage and enrichment of top 1% prioritized non-coding variants in non-exon bases

		% Overlap				Fold Enrichment			
Prioritization Score	% Non-exon	PhastCons	SiPhy-Omega	SiPhy-pi	GERP++	PhastCons	SiPhy-Omega	SiPhy-pi	GERP++
CADD	0.85	96.2	95.0	94.3	98.7	23.9	30.3	20.7	17.8
PhastCons	1.32	100.0	86.5	85.7	93.8	24.8	27.6	18.8	16.9
Eigen	0.77	91.7	89.2	90.0	93.2	22.7	28.5	19.8	16.8
REMM	0.81	88.6	88.4	89.9	95.4	21.9	28.2	19.7	17.2
FATHMM	0.74	86.2	77.5	80.6	85.1	21.4	24.7	17.7	15.3
GERP++	0.84	82.3	78.2	83.4	88.9	20.4	24.9	18.3	16.0
phyloP	0.76	80.8	67.2	70.1	76.4	20.0	21.4	15.4	13.8
LINSIGHT	0.85	70.3	67.6	74.8	74.8	17.4	21.6	16.4	13.5
funSeq2	0.80	18.0	18.4	24.1	23.6	4.5	5.9	5.3	4.2
fitCons	0.42	13.4	13.6	18.2	18.1	3.3	4.3	4.0	3.3
Eigen_PC	0.75	11.0	12.1	16.8	16.3	2.7	3.9	3.7	2.9
CDTS	0.78	10.4	10.1	15.0	15.0	2.6	3.2	3.3	2.7
DANN	0.90	11.0	6.2	6.5	6.7	2.7	2.0	1.4	1.2
FIRE	0.81	1.4	1.0	2.3	2.2	0.3	0.3	0.5	0.4
% Non-Exons		4.0	3.1	4.6	5.6	4.0	3.1	4.6	5.6

Supplementary Figure 3: Constrained non-exonic elements and variant prioritization scores. The figure shows the enrichment in constrained elements for bases that were among the top 1% of prioritized non-coding bases by different scores used for prioritizing genetic variants restricted to non-exonic regions. We restricted to non-exonic regions as exons are already well annotated and CNEP is trained to predict constraint in non-exonic regions. The set of 14 different variant prioritization scores and top 1% non-coding bases was taken from Ref. 1. The rows correspond to different variant prioritization scores. The first column after the name of the variant prioritization scores is the percent of top 1% of non-coding bases for that score is within non-exonic regions. The percentages are not exactly 1% because of ties in the score and some non-coding bases fall within exons. The next four columns report the percent overlap of the prioritized bases with non-exonic bases for four different constrained element set annotations used with CNEP. The final four columns report the fold enrichment of the overlap within non-exonic regions. The bottom row reports the percent of non-exonic genome bases of each of the four constrained element sets cover. This figure highlights how for a number of different variant prioritization scores the top prioritized bases within non-exonic regions heavily enrich for constrained elements including some scores that consider a diverse set of genomic annotations. Source data are provided as a Source Data file.

a

	CNEP (average)	CNEP SiPhy-PI only	CNEP SiPhy-omega only	CNEP PhastCons only	CNEP GERP++ only
CNEP (average)		0.96	0.98	0.96	0.98
CNEP SiPhy-PI only			0.93	0.88	0.91
CNEP SiPhy-omega only				0.93	0.93
CNEP PhastCons only					0.92
CNEP GERP++ only					

b

	Combined Proportion	SiPhy-PI binary label	SiPhy-omega binary label	PhastCons binary label	GERP++ binary label
Combined Proportion		0.84	0.87	0.79	0.85
SiPhy-PI binary label			0.72	0.51	0.60
SiPhy-omega binary label				0.61	0.64
PhastCons binary label					0.55
GERP++ binary label					

Supplementary Figure 4: CNEP score correlations. (a) The panel shows the genome-wide pairwise pearson correlations between the predictions based on applying the CNEP method to each constrained element individually and the CNEP score. The CNEP score was determined based on averaging predictions based on training on each of the four constrained element sets separately. **(b)** The panel shows the genome-wide pairwise pearson correlations computed directly based on the constrained element labels. These were computed by first encoding the value of ‘1’ if a base was a non-exonic base that overlapped a constrained element of the set and a 0 otherwise. Correlations between different element sets were computed directly based on this encoding. Additionally a combined track was computed as the proportion of the four constrained element sets that overlapped a base, and the correlation of each individual set with this combined track was computed. Source data are provided as a Source Data file.

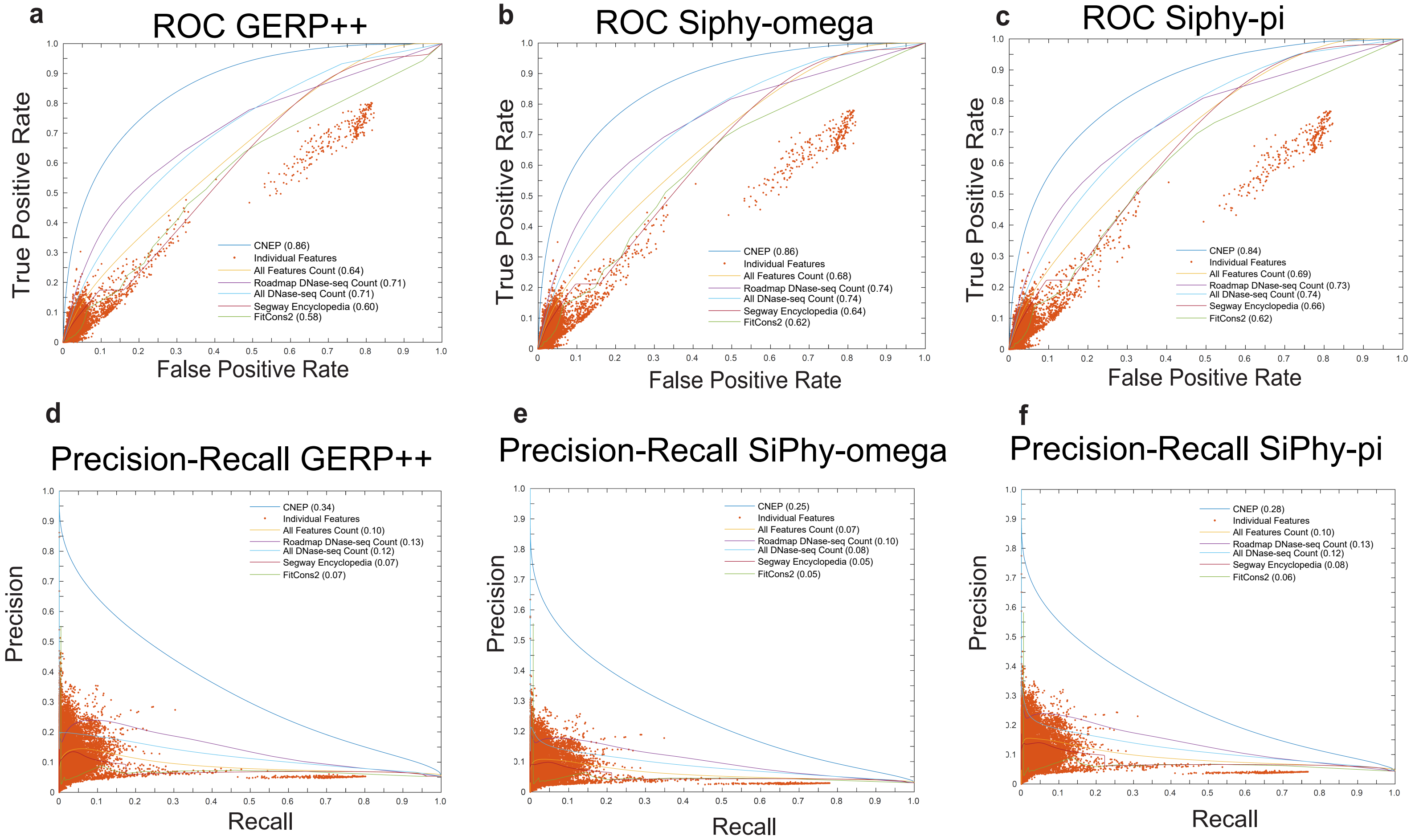

Supplementary Figure 5: ROC and precision-recall curves. This is an extended version of **Fig. 2e,f**. **(a-c)** The receiver operating characteristic (ROC) curves for the CNEP score and other baseline and existing scores for predicting non-exonic bases in constrained elements called by **(a)** GERP++, **(b)** SiPhy-omega, **(c)** SiPhy-pi. **(d-f)** The precision recall curves for the CNEP score and other baseline and existing scores for predicting non-exonic bases in constrained elements called by **(d)** GERP++, **(e)** SiPhy-omega, **(f)** SiPhy-pi. Area under the curve values are shown in parentheses. Source data are provided as a Source Data file.

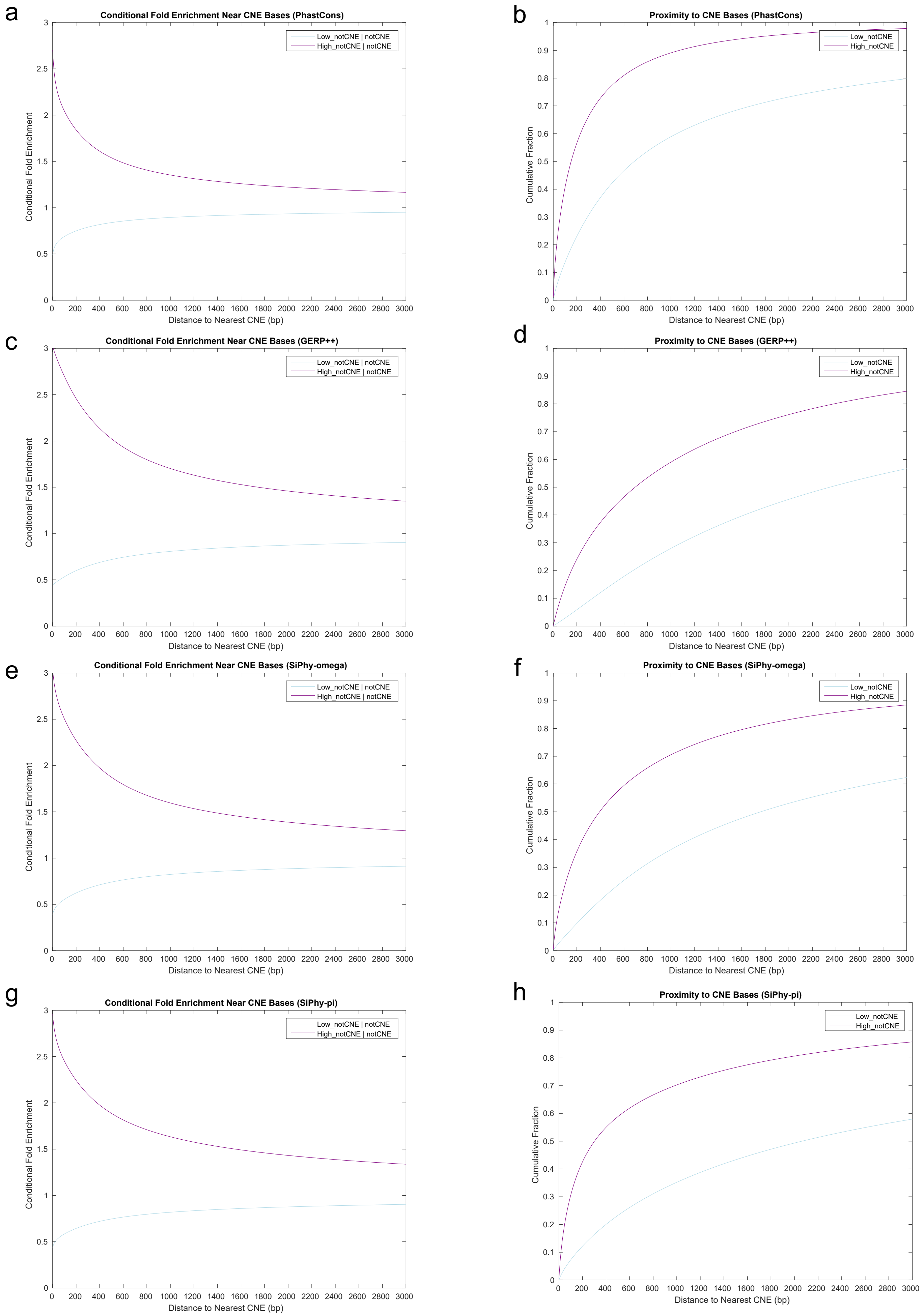

Supplementary Figure 6: Spatial enrichments relative to CNE bases. (a) The plot shows the fold enrichment for the cumulative number of PhastCons High_notCNE and Low_notCNE bases relative to the enrichment of notCNE bases for being within each distance to the nearest CNE base, up to 3,000bp. **(b)** The plot shows the cumulative fraction of PhastCons High_notCNE and Low_notCNE bases at each distance to the nearest CNE base up to 3,000 bp. **(c-h)** The same plots for other constrained element sets: **(c,d)** GERP++, **(e,f)** SiPhy-omega, and **(g,h)** SiPhy-pi. Source data are provided as a Source Data file.

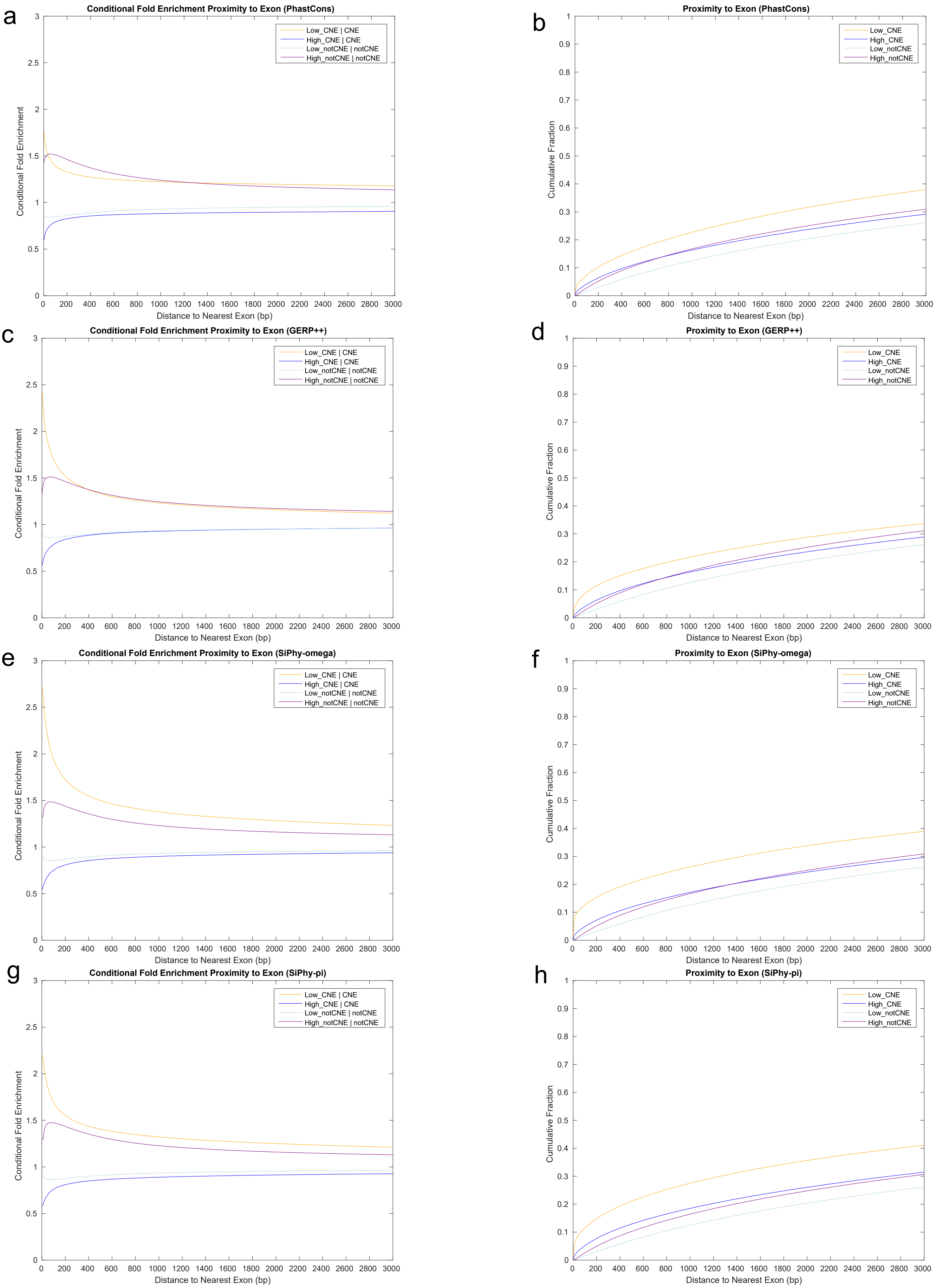

Supplementary Figure 7: Spatial enrichments relative to exons. (a) The plot shows the fold enrichment for the cumulative number of PhastCons Low_CNE and High_CNE bases relative to CNE bases for being within each distance to the nearest exon, up to 3,000bp, and similarly for Low_notCNE and High_notCNE relative to the enrichment of notCNE bases. **(b)** The plot shows the cumulative fraction of PhastCons Low_CNE, High_CNE, Low_notCNE, and High_notCNE bases at each distance to the nearest exon, up to 3,000bp. **(c-h)** The same plots for other constrained element sets: **(c,d)** GERP++, **(e,f)** SiPhy-omega, and **(g,h)** SiPhy-pi. Source data are provided as a Source Data file.

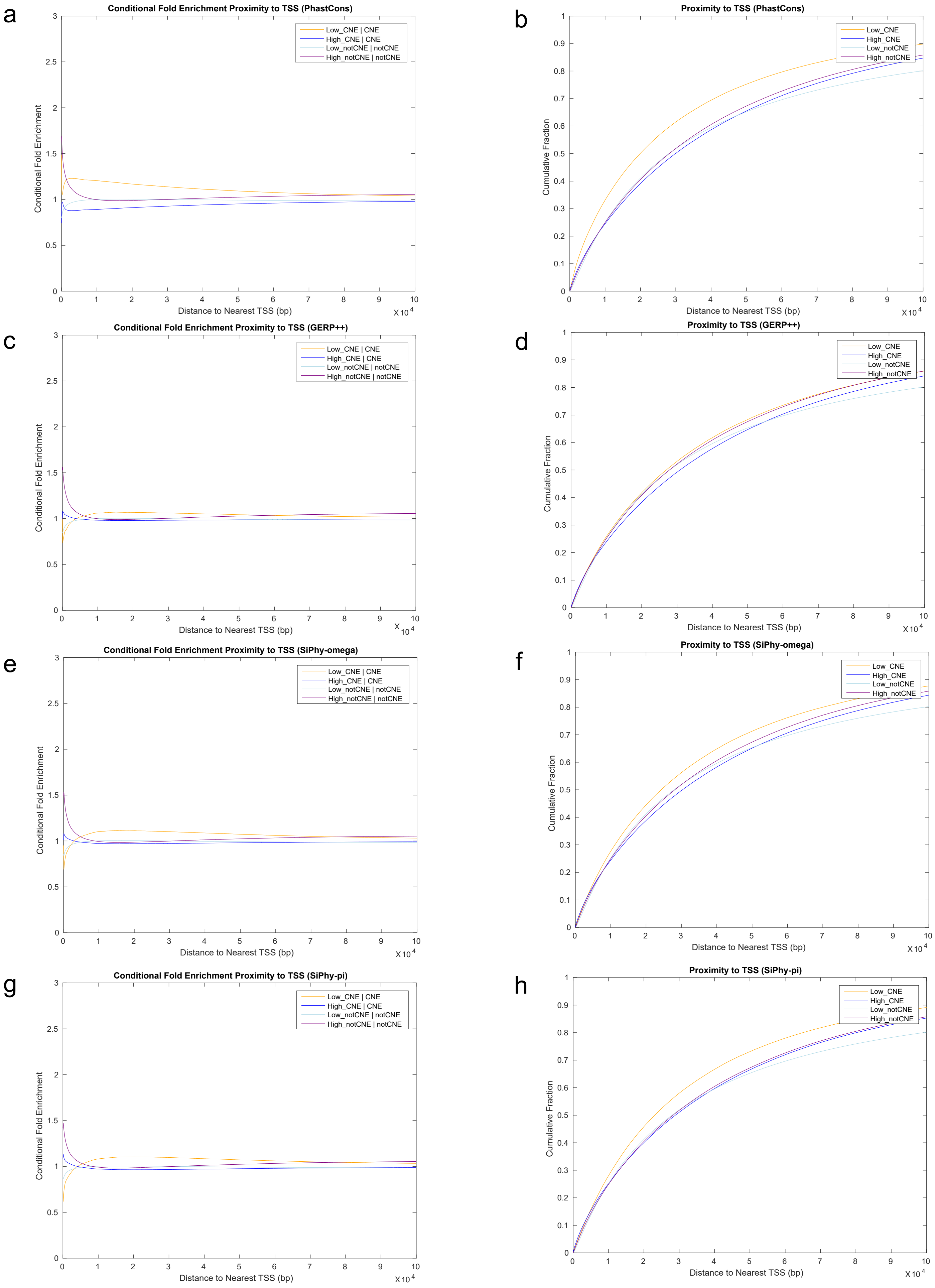

Supplementary Figure 8: Spatial enrichments relative to transcription start sites. (a) The plot shows the fold enrichment for the cumulative number of PhastCons Low_notCNE and High_notCNE bases relative to the enrichment of notCNE bases for being within each distance to a transcription start site, up to 100kb, and similarly for Low_CNE and High_CNE bases relative to the enrichment of CNE bases. **(b)** The plot shows the cumulative fraction of PhastCons Low_CNE, High_CNE, Low_notCNE, and High_notCNE bases at each distance to the nearest transcription start site, up to 100kb. **(c-h)** The same plots for other constrained element sets: **(c,d)** GERP++, **(e,f)** SiPhy-omega, and **(g,h)** SiPhy-pi. Source data are provided as a Source Data file.

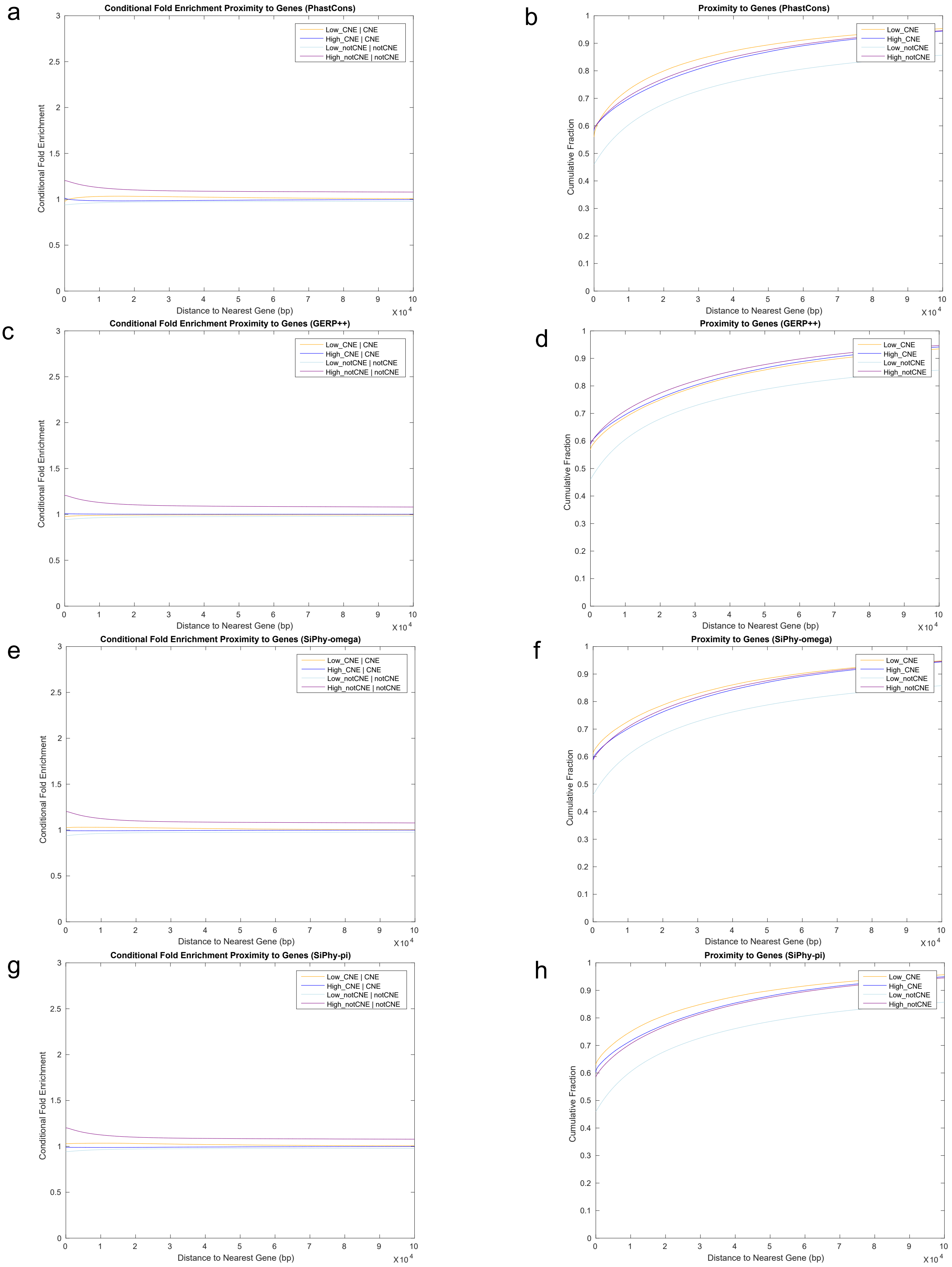

Supplementary Figure 9: Spatial enrichments relative to genes. (a) The plot shows the fold enrichment for the cumulative number of PhastCons Low_notCNE and High_notCNE bases relative to the enrichment of notCNE bases being within each distance to a gene, up to 100kb, and similarly for Low_CNE and High_CNE bases relative to the enrichment of CNE bases. **(b)** The plot shows the cumulative fraction of PhastCons Low_CNE bases at each distance to a gene, up to 100kb. **(c-h)** The same plots for other constrained element sets: **(c,d)** GERP++, **(e,f)** SiPhy-omega, and **(g,h)** SiPhy-pi. Source data are provided as a Source Data file.

a

state	Genome %	CNE				High_CNE			
		PhastCons	SiPhy-Omega	SiPhy-PI	GERP++	PhastCons	SiPhy-Omega	SiPhy-PI	GERP++
1_TssA	0.18	3.03	4.17	3.88	3.07	4.36	5.00	4.89	3.79
2_PromU	0.41	2.10	2.86	2.93	2.24	2.87	3.30	3.49	2.67
3_PromD1	0.41	1.99	2.83	2.94	2.28	2.75	3.24	3.48	2.72
4_PromD2	0.19	1.43	1.66	1.71	1.84	1.70	1.74	1.85	1.98
5_Tx5	2.22	0.95	0.74	0.78	0.81	0.74	0.64	0.67	0.74
6_Tx	0.70	0.71	0.77	0.87	0.70	0.52	0.54	0.61	0.53
7_Tx3	3.48	0.67	0.72	0.90	0.62	0.43	0.46	0.56	0.43
8_TxWk	5.88	0.94	0.80	0.86	0.81	0.75	0.69	0.74	0.73
9_TxReg	0.30	1.98	2.74	2.73	2.42	2.66	3.07	3.15	2.83
10_TxEnh5'	0.38	1.40	1.80	1.99	1.59	1.69	1.88	2.07	1.74
11_TxEnh3'	0.21	0.85	1.20	1.48	0.98	0.85	1.04	1.23	0.88
12_TxEnhW	0.51	1.24	1.35	1.46	1.37	1.34	1.34	1.44	1.42
13_EnhA1	0.22	2.35	3.13	3.20	2.70	3.22	3.64	3.80	3.22
14_EnhA2	0.34	2.61	3.44	3.54	2.98	3.65	4.03	4.22	3.64
15_EnhAF	0.48	1.74	2.24	2.41	1.95	2.26	2.47	2.67	2.26
16_EnhW1	0.28	2.21	2.90	2.93	2.38	3.00	3.34	3.46	2.83
17_EnhW2	0.95	1.77	2.24	2.38	1.97	2.31	2.48	2.61	2.27
18_EnhAc	0.27	1.84	2.25	2.24	2.07	2.41	2.57	2.62	2.42
19_DNase	0.63	2.69	3.44	3.26	2.92	3.71	4.06	3.98	3.51
20_ZNF/Rpts	0.18	0.48	0.19	0.15	0.15	0.07	0.04	0.04	0.04
21_Het	0.91	0.42	0.10	0.10	0.10	0.06	0.04	0.03	0.04
22_PromP	0.20	2.23	2.66	2.46	2.47	2.96	3.08	2.92	2.90
23_PromBiv	0.25	2.61	3.52	3.38	2.86	3.63	4.08	4.09	3.43
24_ReprPC	1.32	1.54	1.85	1.80	1.52	1.89	1.98	1.93	1.68
25_Quies	78.38	0.94	0.90	0.89	0.94	0.92	0.90	0.88	0.92
Base	100	3.87	3.01	4.37	5.33	2.53	2.38	3.25	4.08

state	Low_CNE				notCNE			
	PhastCons	SiPhy-Omega	SiPhy-PI	GERP++	PhastCons	SiPhy-Omega	SiPhy-PI	GERP++
1_TssA	0.54	1.01	0.96	0.72	0.54	0.52	0.48	0.49
2_PromU	0.64	1.21	1.30	0.83	0.79	0.77	0.74	0.75
3_PromD1	0.55	1.30	1.38	0.84	0.64	0.62	0.58	0.60
4_PromD2	0.91	1.40	1.32	1.35	0.87	0.87	0.85	0.84
5_Tx5	1.33	1.13	1.09	1.06	1.02	1.03	1.03	1.03
6_Tx	1.07	1.66	1.61	1.27	0.84	0.83	0.83	0.84
7_Tx3	1.13	1.73	1.85	1.25	0.82	0.82	0.82	0.83
8_TxWk	1.29	1.22	1.24	1.07	0.97	0.97	0.97	0.97
9_TxReg	0.67	1.43	1.49	1.16	0.83	0.81	0.78	0.78
10_TxEnh5'	0.82	1.43	1.67	1.13	0.95	0.94	0.92	0.93
11_TxEnh3'	0.84	1.84	2.15	1.30	0.75	0.74	0.72	0.74
12_TxEnhW	1.00	1.38	1.48	1.23	0.98	0.98	0.97	0.97
13_EnhA1	0.68	1.31	1.56	0.97	0.90	0.89	0.85	0.86
14_EnhA2	0.68	1.23	1.47	0.95	0.91	0.90	0.86	0.86
15_EnhAF	0.77	1.30	1.64	0.98	0.96	0.95	0.92	0.93
16_EnhW1	0.73	1.21	1.33	0.90	0.92	0.91	0.88	0.88
17_EnhW2	0.79	1.30	1.60	0.99	0.95	0.95	0.92	0.93
18_EnhAc	0.80	1.14	1.29	0.96	0.96	0.95	0.94	0.93
19_DNase	0.74	1.14	1.19	0.87	0.93	0.92	0.89	0.89
20_ZNF/Rpts	1.26	0.70	0.48	0.49	0.84	0.85	0.86	0.87
21_Het	1.09	0.34	0.29	0.32	1.02	1.02	1.04	1.05
22_PromP	0.87	1.07	1.05	0.98	0.86	0.86	0.85	0.83
23_PromBiv	0.66	1.32	1.32	0.97	0.73	0.72	0.69	0.69
24_ReprPC	0.89	1.37	1.40	0.98	0.93	0.93	0.92	0.92
25_Quies	0.98	0.92	0.90	0.99	1.02	1.02	1.03	1.02
Base	1.34	0.63	1.12	1.25	92.13	92.99	91.63	90.67

state	High_notCNE				Low_notCNE			
	PhastCons	SiPhy-Omega	SiPhy-PI	GERP++	PhastCons	SiPhy-Omega	SiPhy-PI	GERP++
1_TssA	1.70	1.65	1.53	1.61	0.19	0.19	0.18	0.18
2_PromU	1.95	1.91	1.82	1.92	0.43	0.42	0.42	0.42
3_PromD1	1.81	1.76	1.66	1.74	0.28	0.27	0.26	0.27
4_PromD2	1.75	1.75	1.72	1.69	0.61	0.61	0.60	0.60
5_Tx5	0.97	0.98	0.99	0.99	1.04	1.04	1.04	1.04
6_Tx	0.88	0.87	0.88	0.90	0.82	0.82	0.81	0.82
7_Tx3	0.79	0.78	0.78	0.82	0.84	0.83	0.83	0.83
8_TxWk	0.91	0.92	0.92	0.93	0.98	0.99	0.98	0.99
9_TxReg	2.11	2.06	2.01	2.03	0.44	0.43	0.43	0.43
10_TxEnh5'	1.74	1.73	1.70	1.74	0.71	0.70	0.69	0.71
11_TxEnh3'	1.11	1.09	1.06	1.12	0.63	0.62	0.61	0.62
12_TxEnhW	1.54	1.54	1.53	1.53	0.81	0.81	0.81	0.81
13_EnhA1	2.10	2.05	1.95	2.02	0.52	0.52	0.51	0.52
14_EnhA2	2.25	2.21	2.11	2.14	0.51	0.51	0.50	0.51
15_EnhAF	1.83	1.80	1.74	1.81	0.69	0.68	0.67	0.69
16_EnhW1	2.01	1.98	1.90	1.97	0.59	0.59	0.58	0.59
17_EnhW2	1.78	1.75	1.69	1.75	0.71	0.70	0.69	0.70
18_EnhAc	1.79	1.78	1.73	1.76	0.70	0.70	0.70	0.70
19_DNase	2.24	2.21	2.15	2.16	0.54	0.54	0.54	0.54
20_ZNF/Rpts	0.18	0.18	0.19	0.19	1.04	1.04	1.05	1.05
21_Het	0.12	0.12	0.13	0.13	1.29	1.29	1.30	1.30
22_PromP	1.85	1.84	1.81	1.77	0.56	0.57	0.56	0.56
23_PromBiv	2.09	2.05	1.96	2.02	0.33	0.32	0.32	0.32
24_ReprPC	1.47	1.46	1.45	1.48	0.78	0.78	0.78	0.78
25_Quies	0.95	0.95	0.96	0.95	1.04	1.04	1.05	1.04
Base	21.14	21.29	20.43	19.59	70.99	71.70	71.21	71.08

b

state	Genome %	Low_CNE/CNE				High_notCNE/notCNE			
		PhastCons	SiPhy-Omega	SiPhy-PI	GERP++	PhastCons	SiPhy-Omega	SiPhy-PI	GERP++
1_TssA	0.18	0.18	0.24	0.25	0.24	3.17	3.15	3.16	3.27
2_PromU	0.41	0.30	0.42	0.45	0.37	2.48	2.47	2.47	2.55
3_PromD1	0.41	0.28	0.46	0.47	0.37	2.84	2.84	2.86	2.92
4_PromD2	0.19	0.64	0.84	0.77	0.73	2.01	2.01	2.02	2.02
5_Tx5	2.22	1.40	1.53	1.40	1.31	0.95	0.95	0.96	0.96
6_Tx	0.70	1.51	2.14	1.86	1.82	1.05	1.05	1.06	1.08
7_Tx3	3.48	1.67	2.39	2.06	2.00	0.96	0.95	0.96	0.98
8_TxWk	5.88	1.37	1.53	1.45	1.33	0.95	0.95	0.95	0.96
9_TxReg	0.30	0.34	0.52	0.54	0.48	2.55	2.54	2.57	2.61
10_TxEnh5'	0.38	0.59	0.79	0.84	0.71	1.84	1.84	1.85	1.87
11_TxEnh3'	0.21	0.99	1.52	1.45	1.33	1.48	1.48	1.48	1.53
12_TxEnhW	0.51	0.81	1.03	1.02	0.90	1.57	1.57	1.58	1.58
13_EnhA1	0.22	0.29	0.42	0.49	0.36	2.34	2.31	2.29	2.36
14_EnhA2	0.34	0.26	0.36	0.41	0.32	2.47	2.45	2.45	2.49
15_EnhAF	0.48	0.44	0.58	0.68	0.50	1.91	1.90	1.89	1.94
16_EnhW1	0.28	0.33	0.42	0.45	0.38	2.19	2.18	2.17	2.22
17_EnhW2	0.95	0.44	0.58	0.68	0.50	1.86	1.85	1.84	1.88
18_EnhAc	0.27	0.44	0.51	0.57	0.46	1.87	1.86	1.85	1.89
19_DNase	0.63	0.28	0.33	0.36	0.30	2.41	2.40	2.41	2.43
20_ZNF/Rpts	0.18	2.61	3.76	3.21	3.39	0.21	0.21	0.22	0.22
21_Het	0.91	2.59	3.49	2.94	3.19	0.12	0.12	0.12	0.12
22_PromP	0.20	0.39	0.40	0.43	0.40	2.14	2.13	2.15	2.14
23_PromBiv	0.25	0.25	0.37	0.39	0.34	2.85	2.84	2.86	2.92
24_ReprPC	1.32	0.58	0.74	0.78	0.64	1.58	1.58	1.58	1.60
25_Quies	78.38	1.05	1.02	1.02	1.05	0.93	0.93	0.93	0.93
Base	100								

Supplementary Figure 10: Enrichments for chromatin states. (a) The heatmaps show the median enrichments for CNE, High_CNE, Low_CNE, notCNE, High_notCNE, and Low_notCNE bases for 25 ChromHMM chromatin states based on 12 marks using imputed data across 127 reference epigenomes[2] The values in the table are fold enrichments computed using ChromHMM. The first column reports the % of the genome each state covers and the bottom row the % of the genome the corresponding column covers. **(b)** The heatmaps show the ratio of the Low_CNE to CNE enrichments and High_notCNE to notCNE enrichments. Source data are provided as a Source Data file.

Repeat Type	CNE				High_CNE				Low_CNE				notCNE				High_notCNE				Low_notCNE				High_notCNE/notCNE			
	PhastCons	SiPhy-Omega	SiPhy-PI	GERP++	PhastCons	SiPhy-Omega	SiPhy-PI	GERP++	PhastCons	SiPhy-Omega	SiPhy-PI	GERP++	PhastCons	SiPhy-Omega	SiPhy-PI	GERP++	PhastCons	SiPhy-Omega	SiPhy-PI	GERP++	PhastCons	SiPhy-Omega	SiPhy-PI	GERP++	PhastCons	SiPhy-Omega	SiPhy-PI	GERP++
repeats - all	0.35	0.17	0.22	0.17	0.19	0.14	0.16	0.14	0.65	0.30	0.39	0.25	1.11	1.11	1.12	1.14	0.62	0.62	0.64	0.66	1.26	1.26	1.26	1.27	0.56	0.56	0.57	0.58
DNA_class	0.64	0.41	0.47	0.41	0.51	0.38	0.41	0.39	0.88	0.56	0.63	0.48	1.10	1.10	1.11	1.12	1.34	1.35	1.39	1.43	1.03	1.03	1.03	1.03	1.22	1.23	1.25	1.28
LINE_class	0.40	0.16	0.19	0.16	0.18	0.11	0.13	0.13	0.80	0.31	0.36	0.27	1.12	1.12	1.13	1.14	0.65	0.65	0.67	0.69	1.26	1.26	1.27	1.27	0.58	0.58	0.59	0.61
Low_complexity_class	1.24	0.84	0.79	0.95	0.92	0.77	0.73	0.87	1.82	1.08	0.96	1.23	1.04	1.05	1.06	1.05	0.90	0.92	0.94	0.92	1.08	1.09	1.09	1.09	0.87	0.88	0.88	0.87
LTR_class	0.20	0.07	0.13	0.06	0.09	0.05	0.07	0.04	0.43	0.17	0.29	0.10	1.12	1.12	1.13	1.15	0.63	0.63	0.65	0.68	1.27	1.26	1.27	1.27	0.56	0.56	0.57	0.59
Other_class	0.14	0.00	0.00	0.00	0.00	0.00	0.00	0.00	0.41	0.00	0.00	0.00	1.12	1.12	1.13	1.15	0.01	0.01	0.01	0.01	1.45	1.45	1.46	1.46	0.01	0.01	0.01	0.01
Satellite_class	0.12	0.01	0.00	0.01	0.02	0.00	0.00	0.00	0.31	0.02	0.01	0.02	1.13	1.12	1.14	1.15	0.09	0.09	0.10	0.10	1.44	1.43	1.44	1.44	0.08	0.08	0.08	0.09
Simple_repeat_class	0.86	0.22	0.27	0.25	0.27	0.18	0.20	0.23	1.97	0.35	0.46	0.30	1.08	1.10	1.11	1.12	0.53	0.54	0.55	0.56	1.24	1.26	1.27	1.27	0.49	0.49	0.49	0.50
SINE_class	0.20	0.14	0.22	0.13	0.12	0.11	0.15	0.11	0.36	0.26	0.42	0.20	1.11	1.11	1.12	1.13	0.40	0.40	0.41	0.43	1.33	1.32	1.32	1.33	0.36	0.36	0.36	0.38
Unknown_class	4.99	6.10	4.96	5.06	6.20	6.57	5.51	5.34	2.71	4.34	3.39	4.15	0.91	0.92	0.89	0.84	1.75	1.74	1.71	1.58	0.66	0.67	0.66	0.64	1.92	1.90	1.91	1.87
Alu_family	0.07	0.00	0.04	0.00	0.00	0.00	0.00	0.00	0.19	0.01	0.14	0.00	1.12	1.11	1.13	1.14	0.04	0.04	0.04	0.04	1.45	1.43	1.44	1.45	0.03	0.03	0.03	0.03
centr_family	0.02	0.00	0.00	0.00	0.00	0.00	0.00	0.00	0.05	0.00	0.00	0.00	1.14	1.13	1.15	1.16	0.06	0.06	0.06	0.07	1.46	1.45	1.46	1.46	0.05	0.05	0.06	0.06
CR1_family	1.97	2.06	1.94	2.16	2.03	1.95	1.87	2.03	1.85	2.48	2.11	2.58	1.04	1.05	1.04	1.01	1.92	1.93	1.94	1.91	0.78	0.78	0.78	0.77	1.84	1.84	1.87	1.88
ERV1_family	0.18	0.02	0.03	0.02	0.06	0.01	0.02	0.02	0.40	0.04	0.07	0.03	1.12	1.12	1.14	1.15	0.54	0.54	0.56	0.58	1.30	1.29	1.30	1.30	0.48	0.48	0.49	0.51
ERVK_family	0.08	0.00	0.00	0.00	0.01	0.00	0.00	0.00	0.21	0.00	0.01	0.00	1.12	1.11	1.13	1.14	0.19	0.19	0.20	0.21	1.40	1.39	1.40	1.40	0.17	0.17	0.17	0.18
ERVL_family	0.23	0.09	0.17	0.07	0.11	0.06	0.09	0.06	0.46	0.21	0.39	0.11	1.12	1.12	1.13	1.15	0.81	0.81	0.84	0.88	1.21	1.21	1.21	1.22	0.72	0.73	0.74	0.77
ERVL-MaLR_family	0.19	0.08	0.15	0.05	0.07	0.04	0.07	0.03	0.43	0.22	0.37	0.11	1.12	1.12	1.13	1.15	0.59	0.59	0.61	0.64	1.28	1.27	1.28	1.28	0.53	0.53	0.54	0.56
Gypsy?_family	0.74	0.72	0.99	0.68	0.64	0.58	0.78	0.59	0.94	1.25	1.60	0.98	1.10	1.10	1.09	1.11	1.58	1.58	1.59	1.66	0.96	0.96	0.95	0.96	1.43	1.44	1.46	1.50
Gypsy_family	0.76	0.65	0.77	0.64	0.62	0.54	0.60	0.56	1.02	1.05	1.25	0.90	1.09	1.09	1.10	1.11	1.35	1.35	1.38	1.42	1.02	1.02	1.01	1.02	1.23	1.24	1.26	1.28
hAT_family	0.55	0.27	0.37	0.19	0.34	0.18	0.26	0.14	0.94	0.62	0.70	0.32	1.09	1.10	1.11	1.12	1.52	1.53	1.57	1.65	0.97	0.97	0.97	0.98	1.39	1.39	1.42	1.47
hAT-Blackjack_family	0.54	0.24	0.37	0.23	0.38	0.18	0.28	0.20	0.84	0.45	0.65	0.34	1.11	1.11	1.12	1.13	1.63	1.64	1.69	1.76	0.95	0.95	0.95	0.96	1.47	1.48	1.51	1.55
hAT-Charlie_family	0.63	0.36	0.47	0.36	0.46	0.29	0.38	0.31	0.95	0.66	0.74	0.51	1.10	1.10	1.11	1.12	1.57	1.58	1.62	1.69	0.95	0.96	0.96	0.96	1.43	1.44	1.47	1.51
hAT-Tip100_family	0.53	0.25	0.35	0.24	0.37	0.19	0.23	0.21	0.85	0.47	0.68	0.34	1.11	1.11	1.12	1.13	1.39	1.40	1.45	1.51	1.02	1.02	1.02	1.03	1.26	1.26	1.30	1.33
L1_family	0.34	0.07	0.08	0.08	0.12	0.04	0.05	0.05	0.77	0.16	0.18	0.15	1.12	1.12	1.14	1.15	0.52	0.52	0.54	0.56	1.30	1.30	1.31	1.31	0.46	0.46	0.47	0.49
L2_family	0.45	0.32	0.49	0.29	0.25	0.20	0.30	0.21	0.83	0.77	1.04	0.54	1.11	1.11	1.11	1.13	1.10	1.09	1.12	1.17	1.11	1.11	1.11	1.12	0.99	0.99	1.01	1.04
MIR_family	0.64	0.57	0.82	0.54	0.48	0.42	0.62	0.45	0.94	1.13	1.39	0.84	1.09	1.09	1.09	1.10	1.72	1.72	1.74	1.82	0.90	0.90	0.90	0.91	1.58	1.58	1.60	1.65
RTE_family	1.75	1.59	1.48	1.87	1.69	1.47	1.42	1.75	1.85	2.06	1.65	2.27	1.05	1.06	1.06	1.03	2.13	2.15	2.19	2.15	0.73	0.74	0.74	0.73	2.02	2.02	2.06	2.08
Satellite_family	0.31	0.03	0.02	0.03	0.06	0.01	0.01	0.01	0.77	0.08	0.04	0.07	1.11	1.11	1.13	1.14	0.16	0.16	0.17	0.18	1.39	1.39	1.40	1.41	0.14	0.15	0.15	0.16
TcMar-Mariner_family	0.70	0.49	0.49	0.62	0.58	0.47	0.48	0.58	0.92	0.56	0.54	0.73	1.07	1.08	1.08	1.08	1.12	1.13	1.16	1.16	1.06	1.06	1.06	1.06	1.05	1.05	1.07	1.07
TcMar-Tc2_family	1.03	0.59	0.55	0.77	0.85	0.50	0.52	0.71	1.37	0.92	0.62	0.96	1.08	1.10	1.11	1.10	1.96	1.99	2.05	2.08	0.82	0.83	0.84	0.83	1.81	1.82	1.86	1.89
TcMar-Tigger_family	0.45	0.27	0.31	0.29	0.33	0.26	0.28	0.28	0.69	0.31	0.39	0.33	1.11	1.11	1.12	1.13	0.96	0.96	0.99	1.02	1.16	1.15	1.16	1.16	0.86	0.87	0.88	0.90

Supplementary Figure 11: Enrichments for repeat elements. The figure shows heatmaps for the enrichments for CNE, High_CNE, Low_CNE, notCNE, High_notCNE, and Low_notCNE bases for each of the constrained element sets for repeats called by RepeatMasker followed by a heatmap for the ratio of the High_notCNE to notCNE enrichments. The first row reports enrichments for all repeats. The following set of rows report enrichment for repeat classes, and then enrichments for repeat families. Only repeat classes or families covering at least a million bases are shown. Source data are provided as a Source Data file.

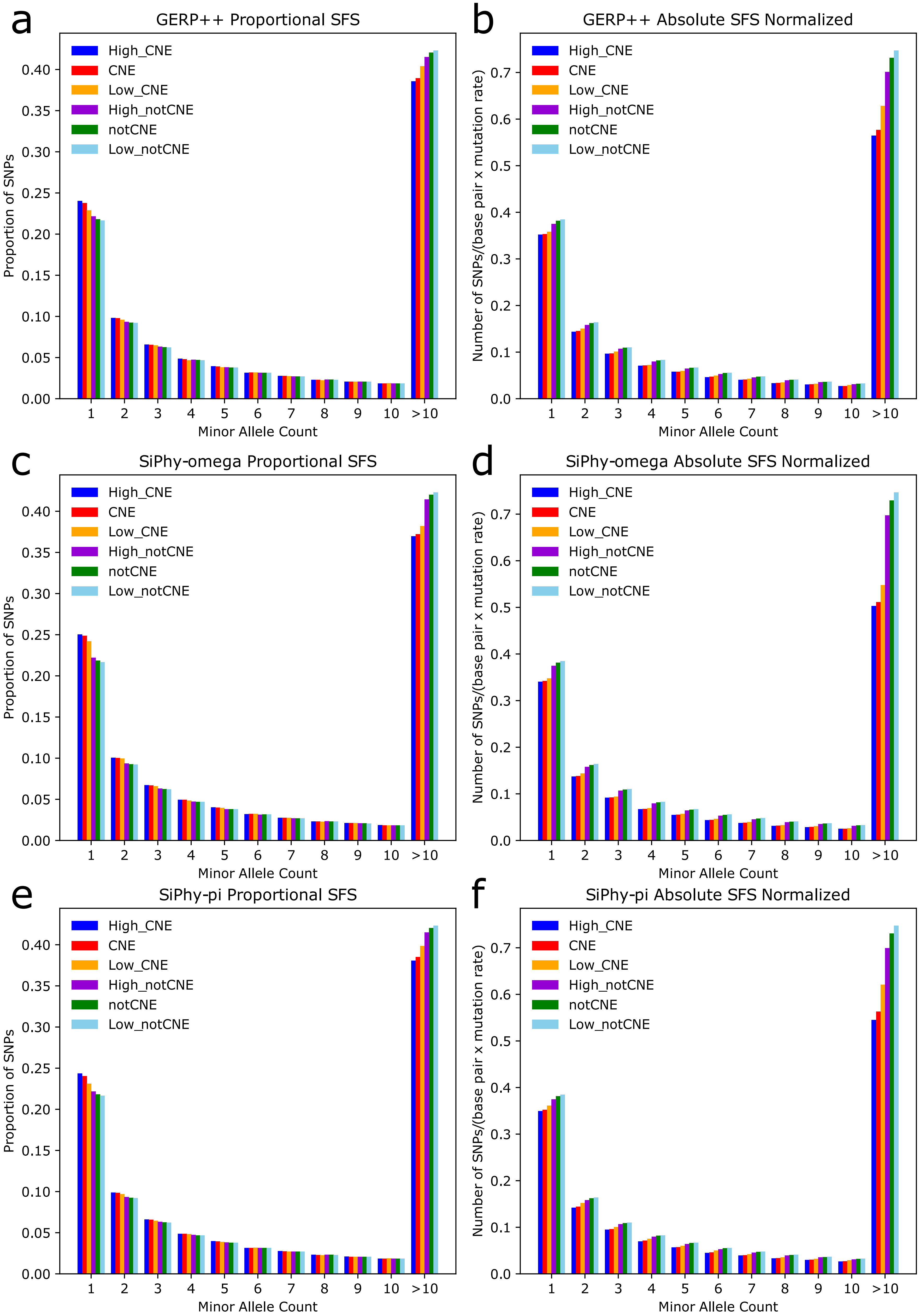

Supplementary Figure 12: Proportional SFS and absolute SFS normalized by average mutation rate. Similar plots as shown in **Fig. 3a,b** except for additional constrained element sets: **(a,b)** GERP++, **(c,d)** SiPhy-omega, and **(e,f)** SiPhy-pi. Source data are provided as a Source Data file.

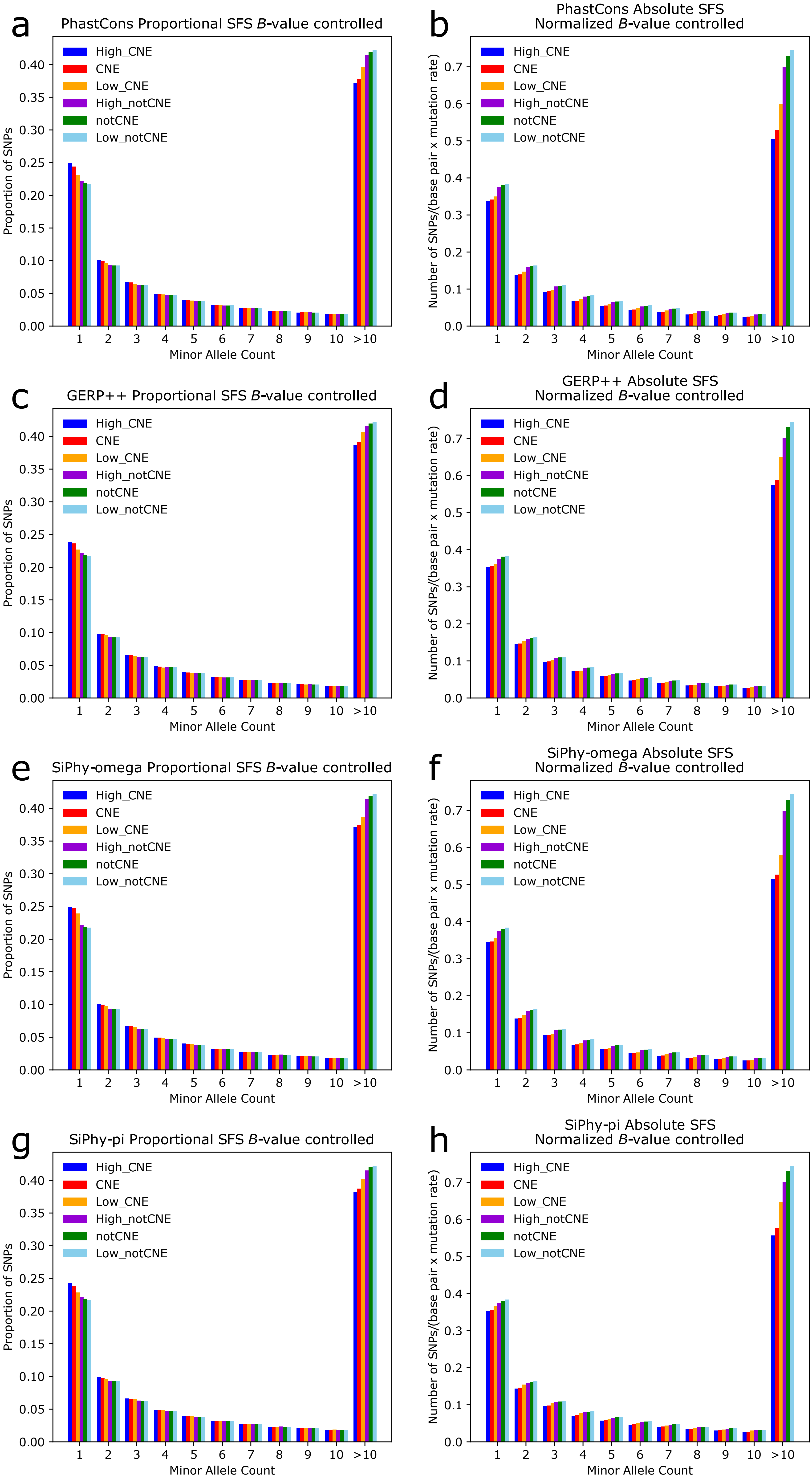

Supplementary Figure 13: SFS analysis controlling for background selection. Similar plots as shown in **Fig. 3a,b** and **Supplementary Fig. 12** except reweighting positions to control for differences in estimated background selection. For the proportional SFS, the weighting is such that the B-value distribution of variants in a set matches the B-value distribution of all variants. For the absolute SFS normalized by mutation rate, the weighting is such that the B-value distributions of all considered non-exonic positions in a set matches the B-value distribution of all considered non-exonic positions. The plots are based on **(a,b)** PhastCons, **(c,d)** GERP++, **(e,f)** SiPhy-omega, and **(g,h)** SiPhy-pi constrained element sets. Source data are provided as a Source Data file.

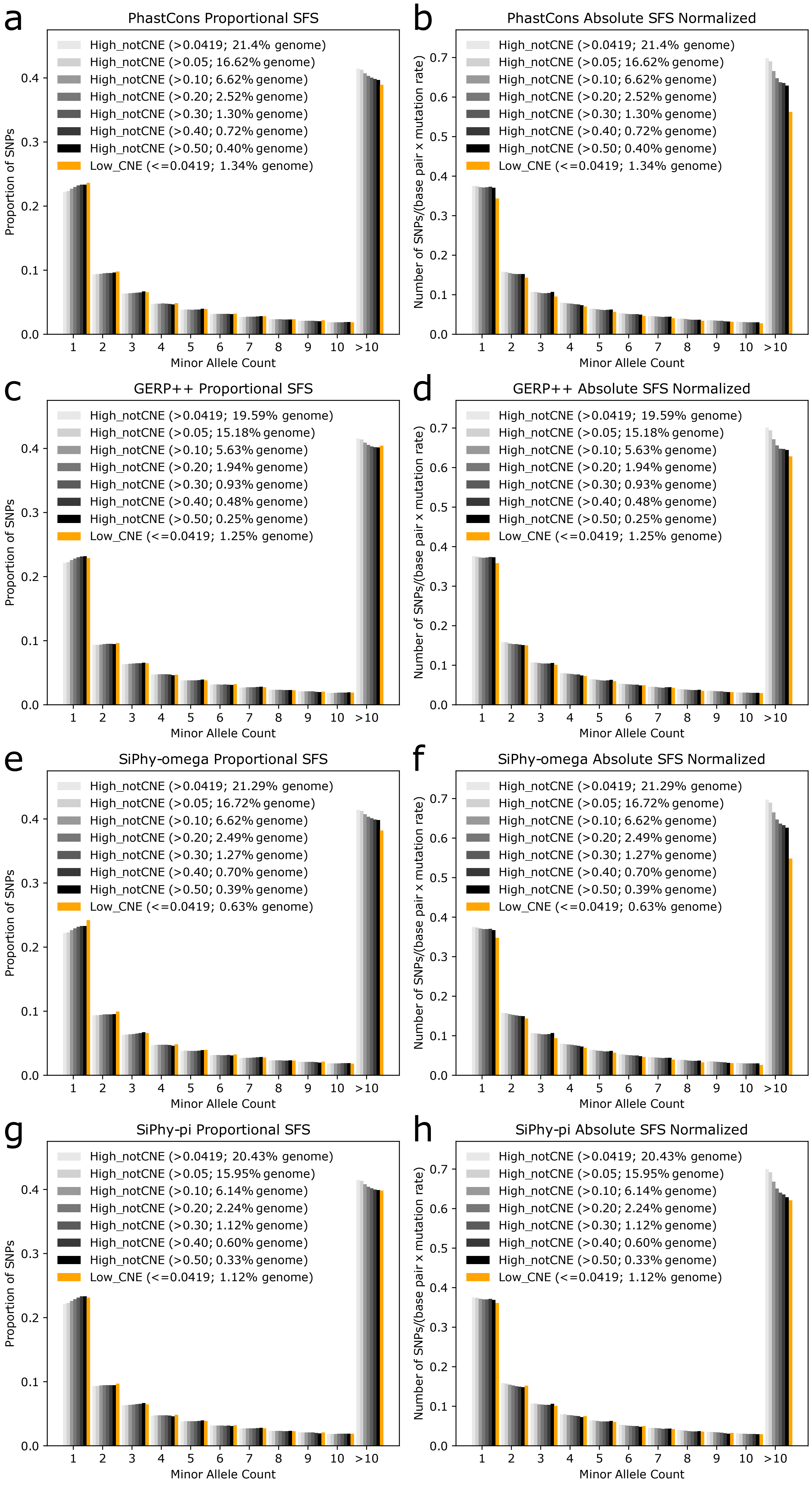

Supplementary Figure 14: Proportional SFS and absolute SFS for subsets of High_notCNE bases. Similar plots as shown in **Fig. 3a,b** and **Supplementary Fig. 12**. The plot shows the proportional SFS and absolute SFS normalized by average mutation rate for High_notCNE bases, which all have a CNEP score >0.0419, and the subsets of High_notCNE bases that have CNEP scores greater than 0.05, 0.10, 0.20, 0.30, 0.40, and 0.50 as indicated in the color legend. Also shown for comparison are the results for the Low_CNE bases, which all have a CNEP score <=0.0419. Plots are shown for **(a,b)** PhastCons, **(c,d)** GERP++, **(e,f)** SiPhy-omega, and **(g,h)** SiPhy-pi. The legend indicates the percent of the genome in each of the sets. Source data are provided as a Source Data file.

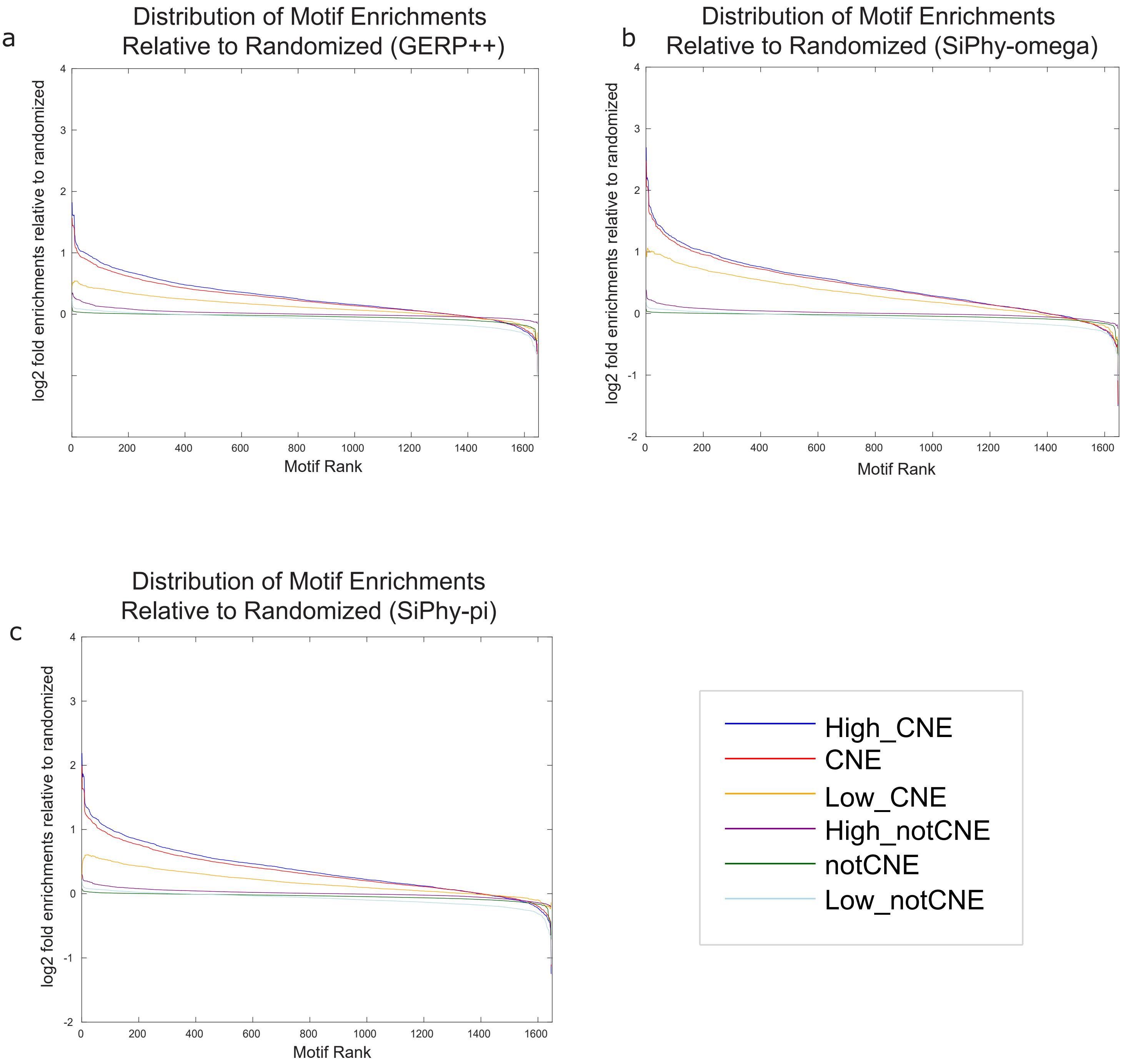

Supplementary Figure 15: Distribution of motif enrichments. Similar plots as shown in **Fig. 4a** except for additional constrained element sets: **(a)** GERP++, **(b)** SiPhy-omega, and **(c)** SiPhy-pi. Source data are provided as a Source Data file.

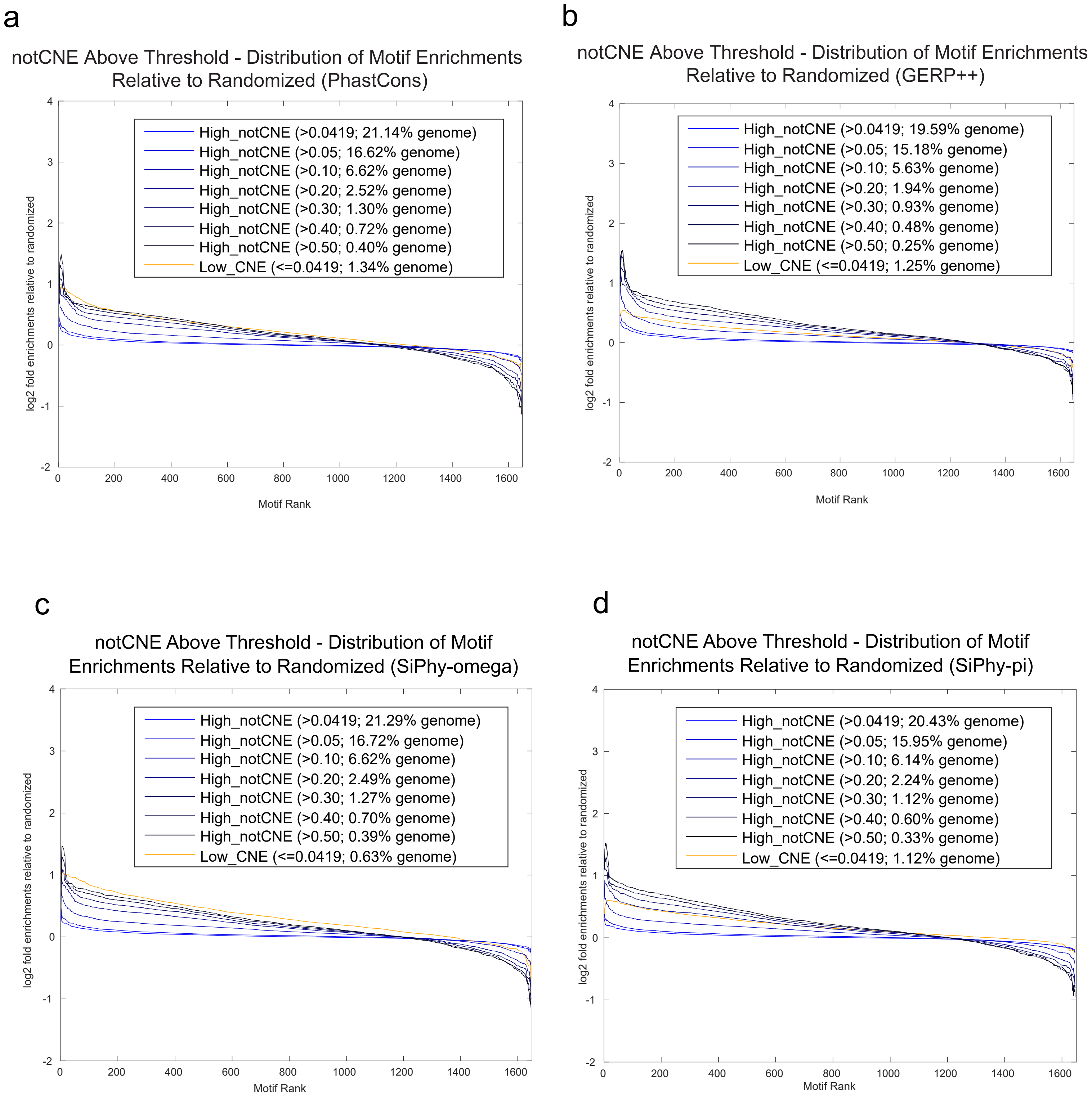

Supplementary Figure 16: Distribution of motif enrichments for subsets of High_notCNE bases. Similar plots to **Fig. 4a** and **Supplementary Fig. 15**. The plot shows the difference of the distribution of motif enrichments relative to the distribution for a randomized set of the motifs for High_notCNE bases, which all have a CNEP score >0.0419, and the subsets of High_notCNE bases that have CNEP scores greater than 0.05, 0.10, 0.20, 0.30, 0.40, and 0.50 as indicated in the color legend. Also shown for comparison are the results for the Low_CNE bases, which all have a CNEP score <=0.0419. The x-axis is the rank position of the motif among the 1,646 motifs and the y-axis is the difference between the $\log_2$ fold enrichment based on the actual motif calls and the median of three randomized versions at the same rank position (Methods), shown for **(a)** PhastCons, **(b)** GERP++, **(c)** SiPhy-omega, and **(d)** SiPhy-pi constrained elements. The legend indicates the percent of the genome in each of the sets. Source data are provided as a Source Data file.

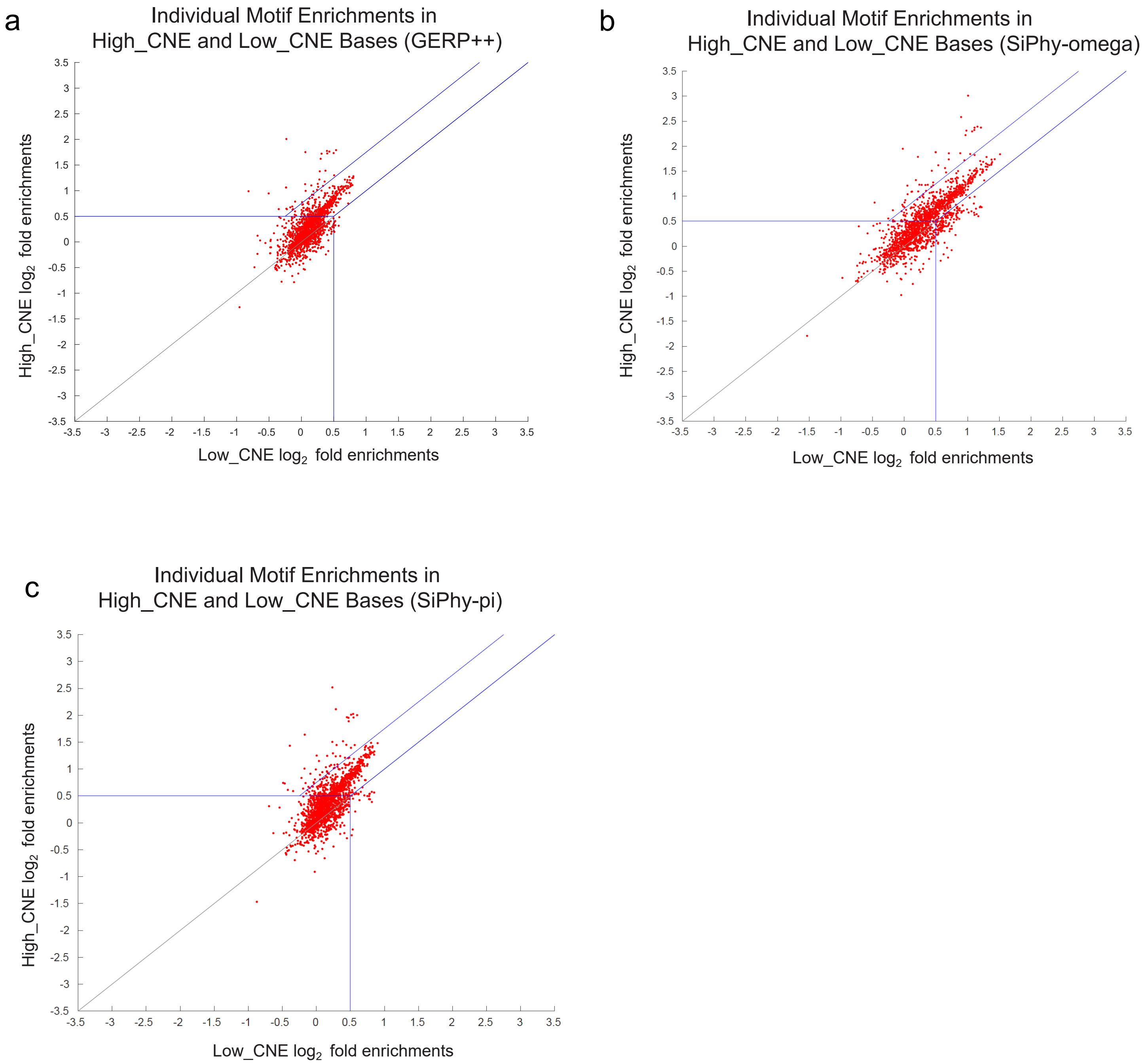

Supplementary Figure 17: Scatter plot of individual motif enrichments. Similar plots as shown in **Fig. 4b** except for additional constrained element sets: **(a)** GERP++, **(b)** SiPhy-omega, and **(c)** SiPhy-pi. The blue lines separate the three regions used for the GO enrichment analysis, 'High_CNE strongly preferred', 'High_CNE moderately preferred', and 'Low_CNE preferred', where at least one of the Low_CNE or the High_CNE $\log_2$ enrichment is greater than or equal to 0.5 (**Supplementary Data 3-4**). Source data are provided as a Source Data file.

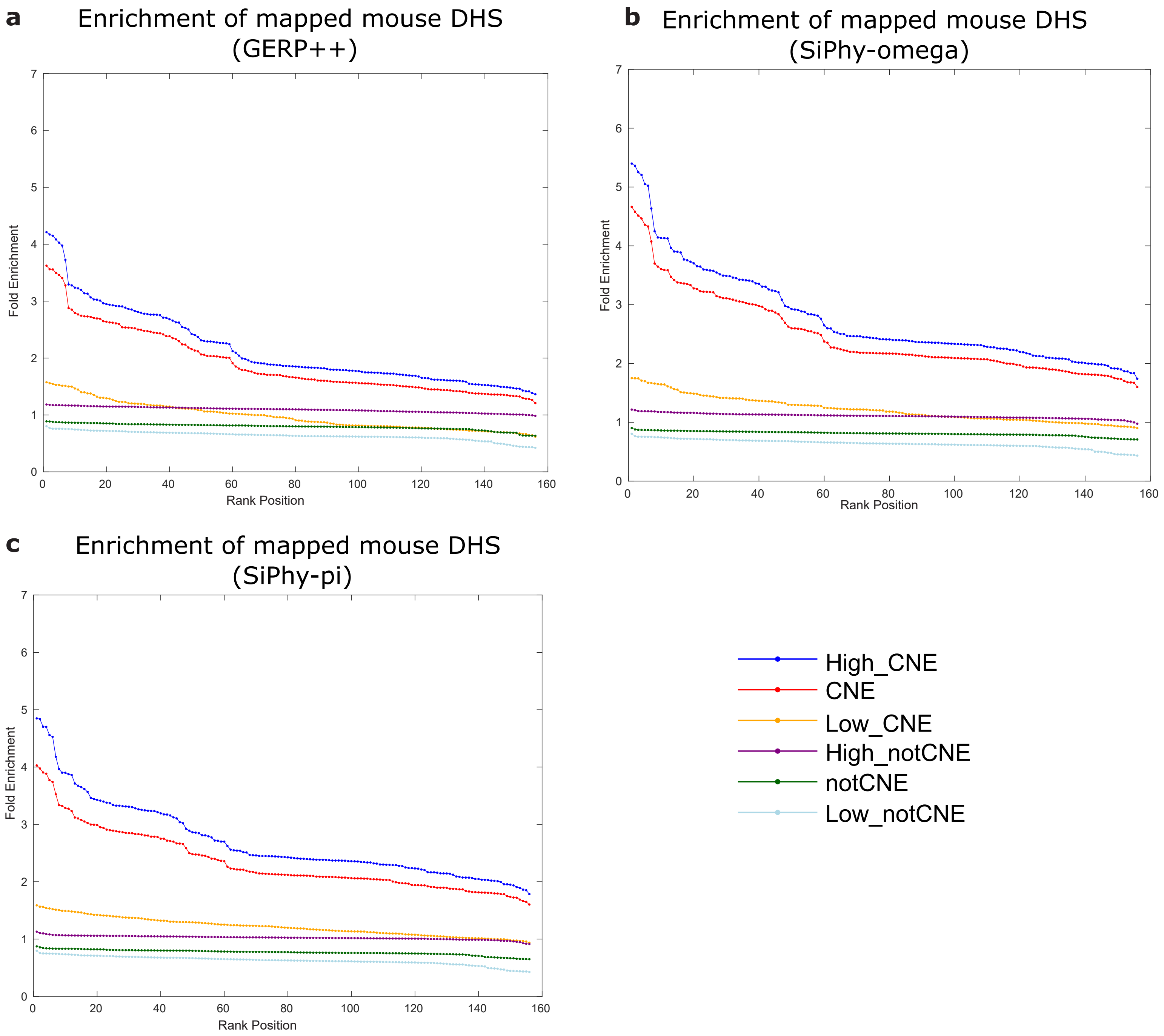

Supplementary Figure 18: Distribution of enrichments for mapped mouse DHS. Similar plots as shown in **Fig. 4c** except for additional constrained element sets: **(a)** GERP++, **(b)** SiPhy-omega, and **(c)** SiPhy-pi. Source data are provided as a Source Data file.

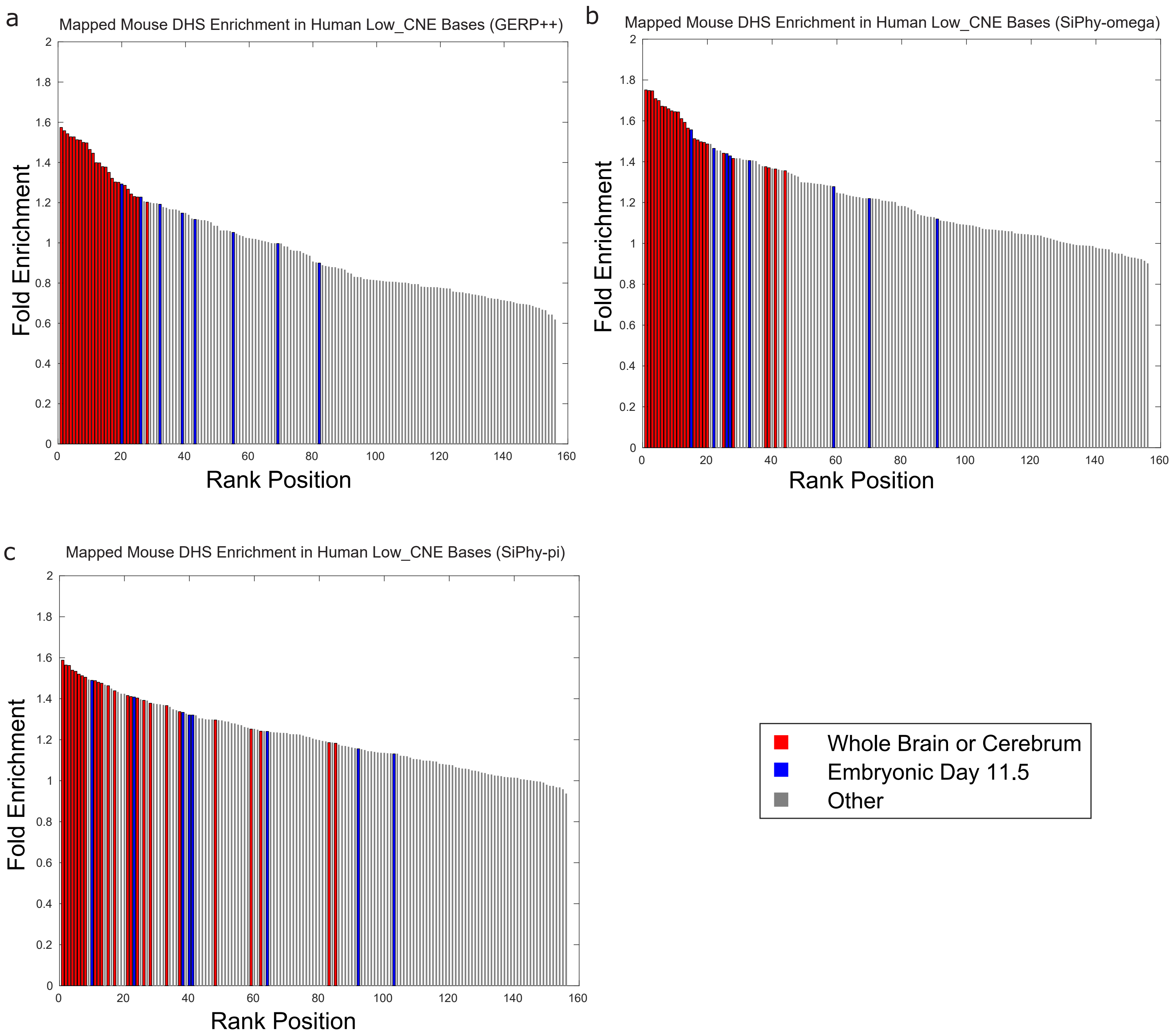

Supplementary Figure 19: **Low_CNE enrichments for mapped mouse DHS.** Similar plots as shown in **Fig. 4d** except for additional constrained element sets: **(a)** GERP++, **(b)** SiPhy-omega, and **(c)** SiPhy-pi. Source data are provided as a Source Data file.

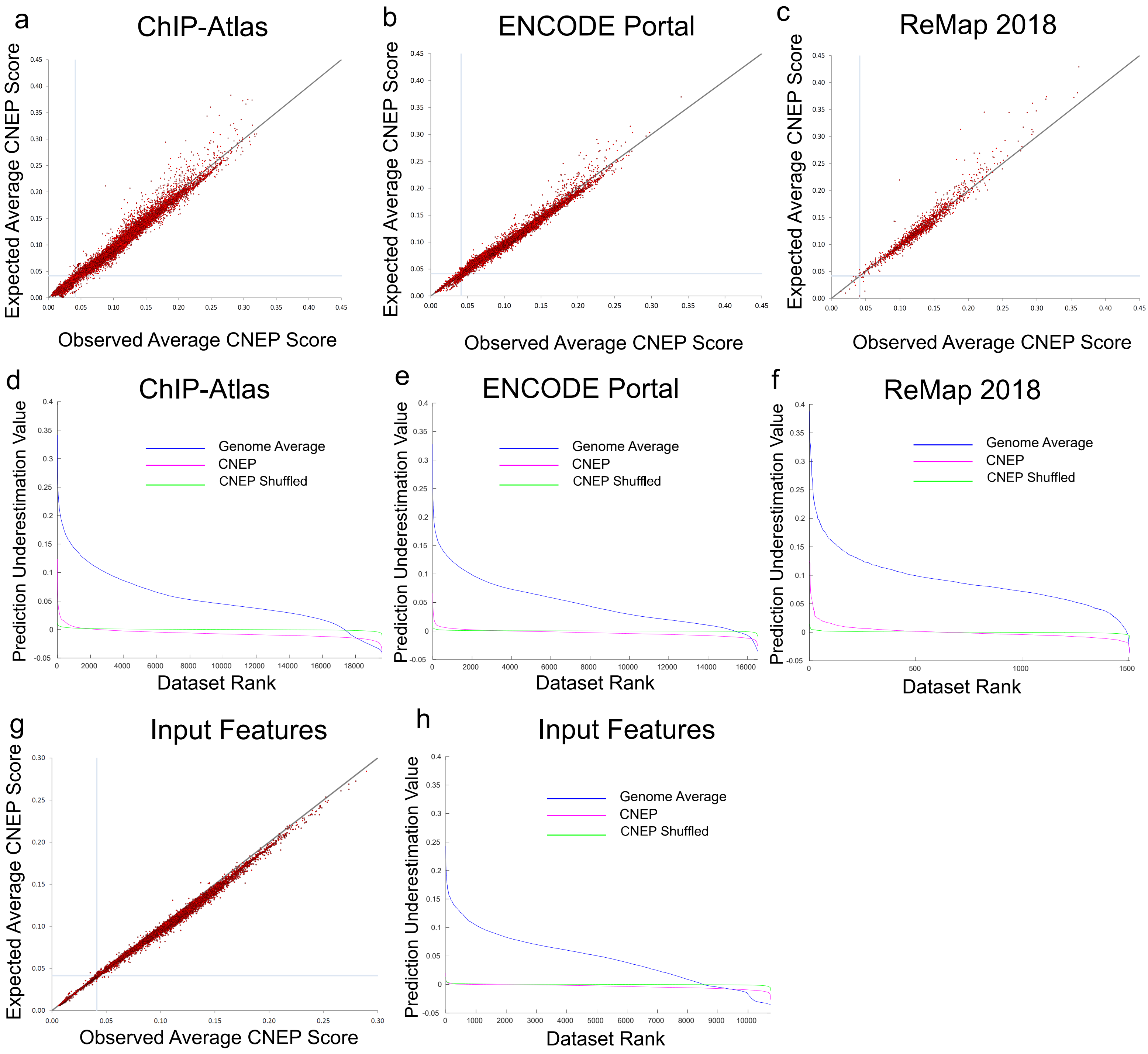

Supplementary Figure 20: Expected vs. observed average CNEP scores. Similar scatter plots as shown in **Fig. 2b** except for **(a)** ChIP-Atlas, **(b)** ENCODE portal, and **(c)** ReMap 2018 datasets using the retrospective CNEP score based on a feature subset available in 2015 (**Methods**). Each point corresponding to one dataset. The x-axis shows the average CNEP score in bases covered by the dataset, while the y-axis shows the expected CNEP score based on the dataset overlap with constrained non-exonic bases. Only datasets that cover at least 200kb are shown. The full table corresponding to these values can be found in **Supplementary Data 6**. The diagonal line is the y=x line. The vertical line corresponds to the genome-wide observed average CNEP score. The horizontal line corresponds to the genome-wide expected average CNEP score. **(d-f)** Plots for **(d)** ChIP-atlas **(e)** ENCODE portal, and **(f)** ReMap 2018 showing the distribution of prediction underestimation values for datasets covering at least 200kb. The prediction underestimation value for a dataset is the average difference between the expected CNEP score and the prediction value for each base the dataset covers. Results are shown for prediction values based on the genome-wide average expected CNEP score (blue) and the CNEP score (magenta). Also shown is the distribution of using the CNEP score for the prediction values, but applied to a shuffled version of each dataset (green). The large gap between the blue and magenta lines suggest that the CNEP score captures most of the marginal information contained in any dataset about the expected frequency of overlap with CNE bases. However, the gap between the magenta and green lines suggest there are some datasets that capture additional marginal information on CNE bases than given by the CNEP score. **(g,h)** Similar plots to (a-c) and (d-f) respectively, but based on the feature subset available in 2015 used for the retrospective predictions. For this, the CNEP score did not have an increase of datasets with a positive CNEP underestimation score compared to shuffles of the datasets. Source data are provided as a Source Data file.

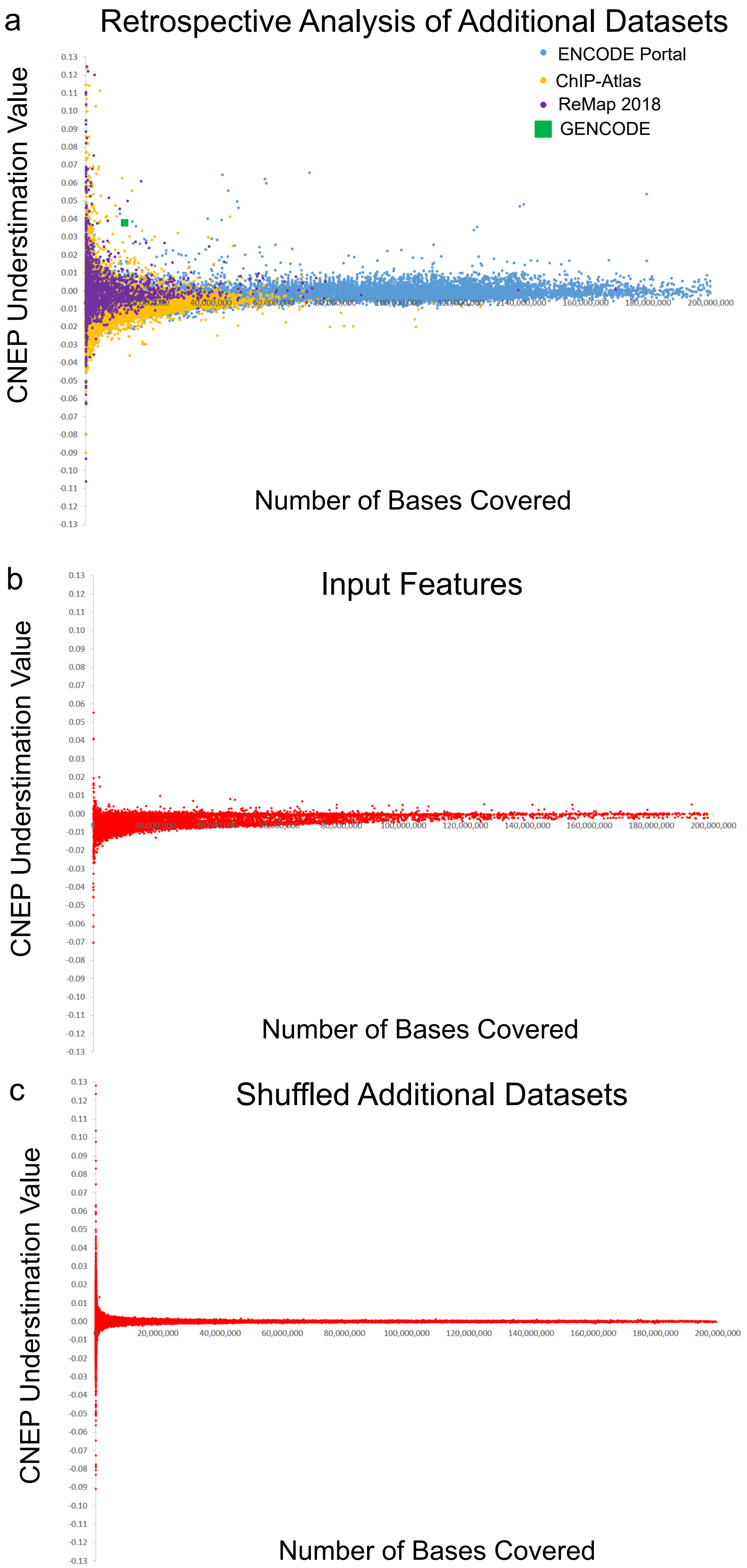

Supplementary Figure 21: CNEP underestimation values in retrospective analysis. (a) Similar plots to **Fig. 5** showing a scatter plot of CNEP underestimation values, but also including negative CNEP underestimation values, meaning the average observed CNEP score for bases covered by the feature is greater than expected based on the features overlap with CNE bases. **(b,c)** Similar to (a) but for the **(b)** input features to CNEP for the retrospective analysis based on features available in 2015 and **(c)** shuffled versions of the additional datasets considered in (a) and **Fig. 5** (**Methods**). Greater underestimation values for the same genomic coverage is seen in (a) than in both these controls, demonstrating additional marginal additive information about CNE bases in the additional datasets. Source data are provided as a Source Data file.

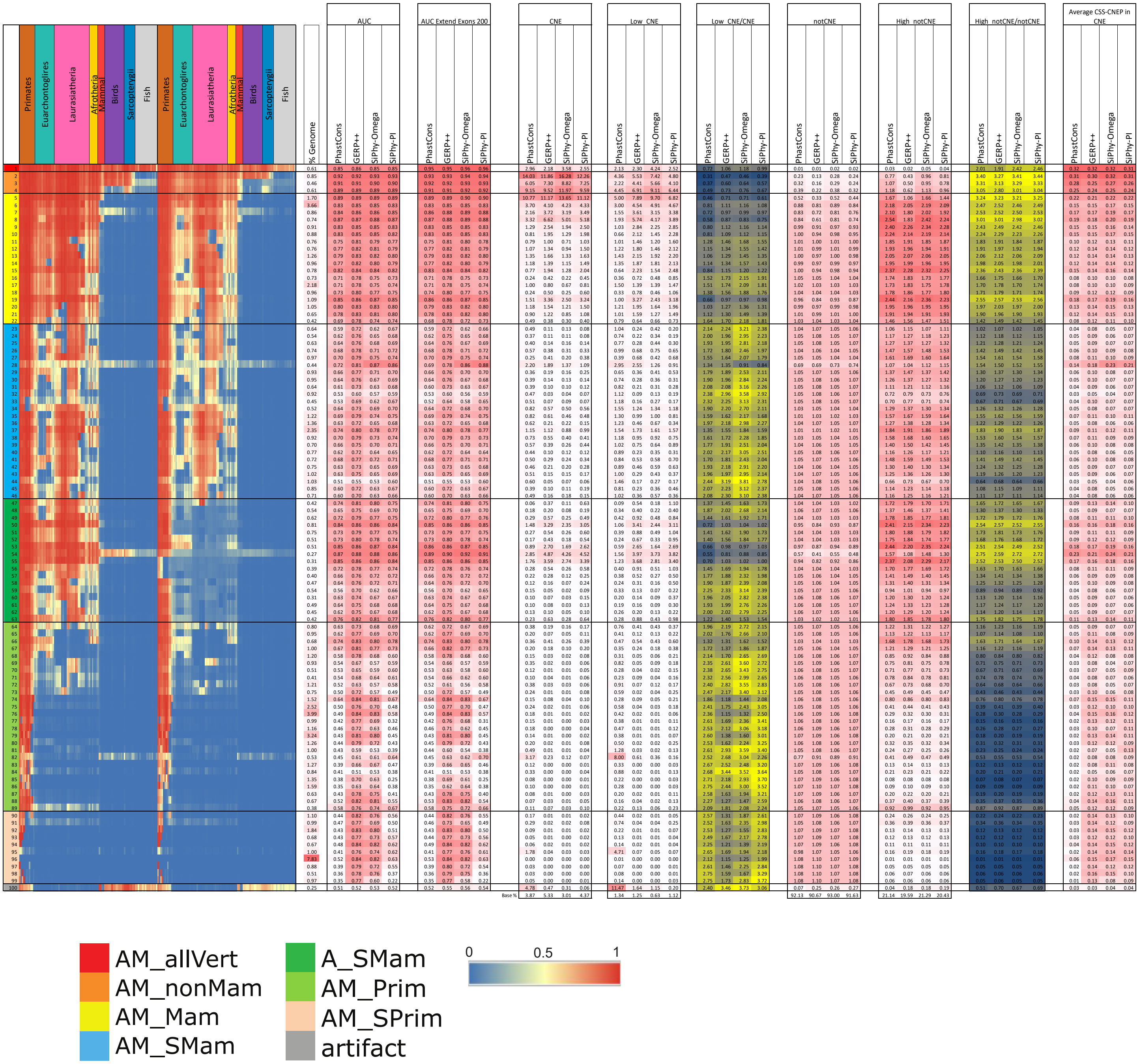

Supplementary Figure 22: Conservation state enrichments. This is an extended version of **Fig. 6a,b** showing the AUC for predicting CNE bases, enrichment values, and average CSS-CNEP score in CNE bases for GERP++, SiPhy-omega, and SiPhy-pi in addition to PhastCons. Source data are provided as a Source Data file.

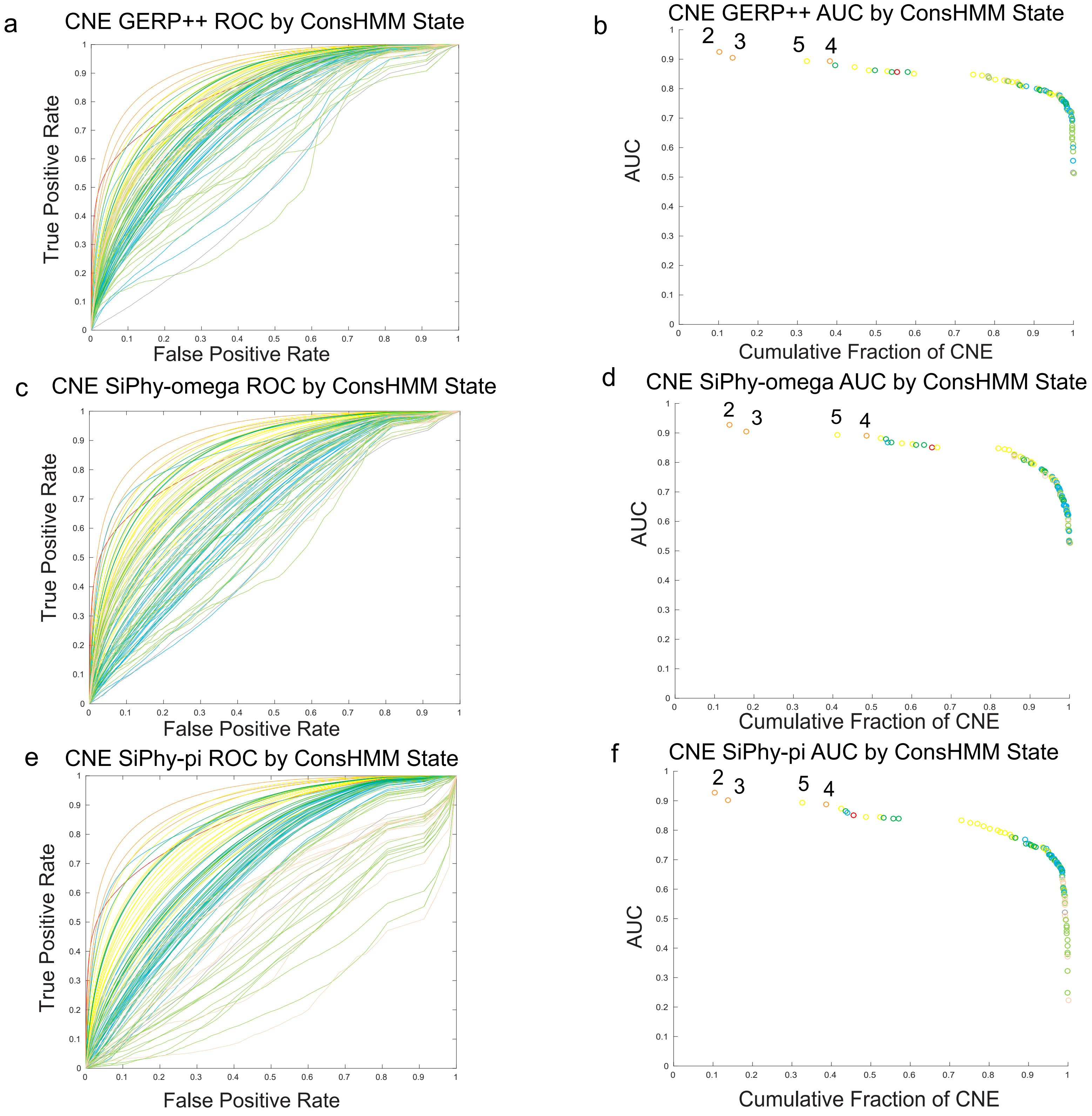

Supplementary Figure 23: Prediction of CNE bases by conservation state. Similar plots as shown in **Fig. 6c,d** except for **(a,b)** GERP++ **(c,d)** SiPhy-omega, and **(e,f)** SiPhy-pi constrained elements. Source data are provided as a Source Data file.

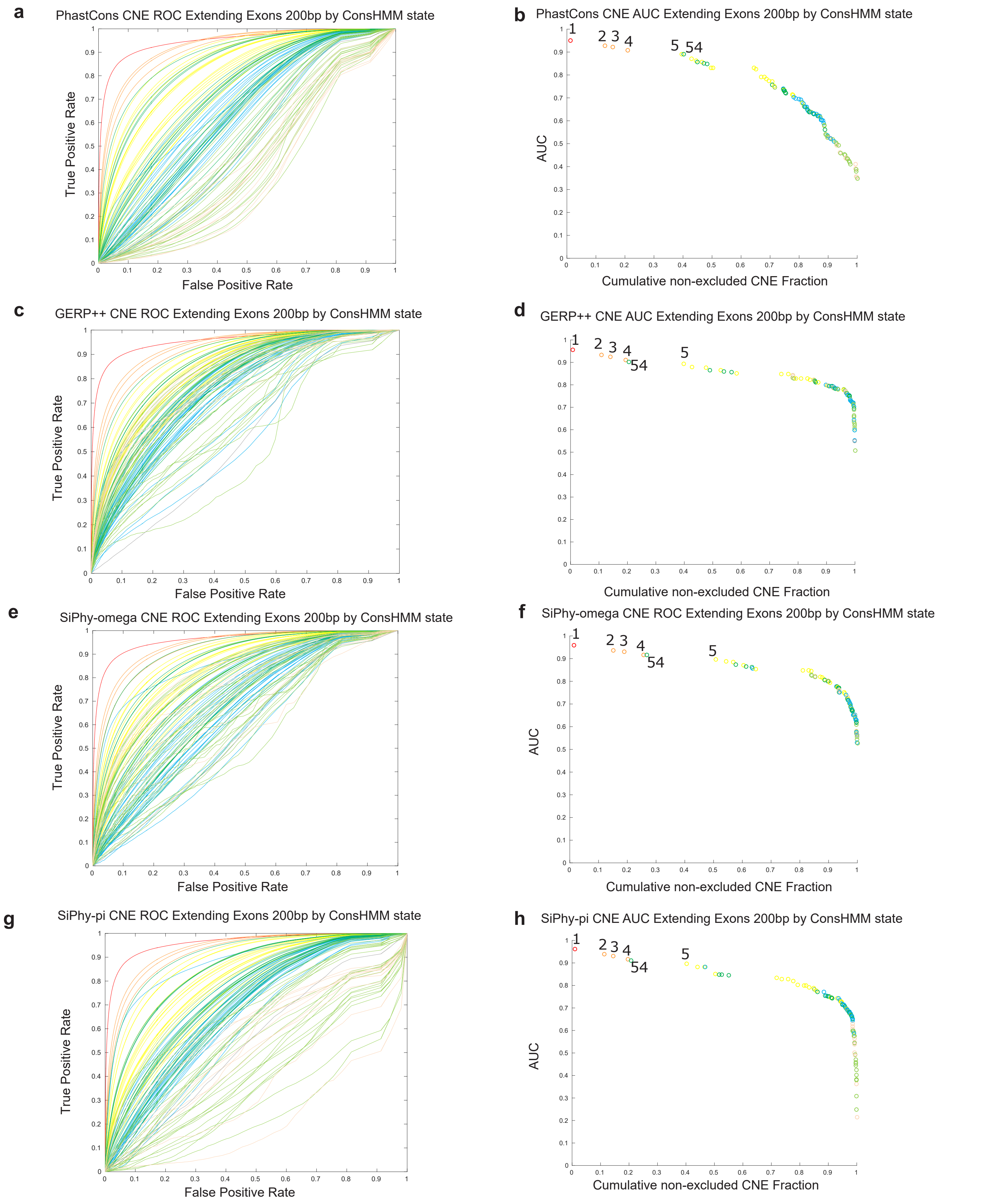

Supplementary Figure 24: Prediction of CNE bases by conservation state extending exons. Similar plots as shown in **Supplementary Fig. 23** and **Fig. 6c,d** except the exon definition is first extended 200bp on each side so that only bases in constrained non-exonic elements more than 200bp from an exon are considered positives. Shown here for all four constrained element sets: **(a,b)** PhastCons, **(c,d)** GERP++, **(e,f)** SiPhy-omega, and **(g,h)** SiPhy-pi. State 1 now becomes the state with the highest AUC overall. Source data are provided as a Source Data file.

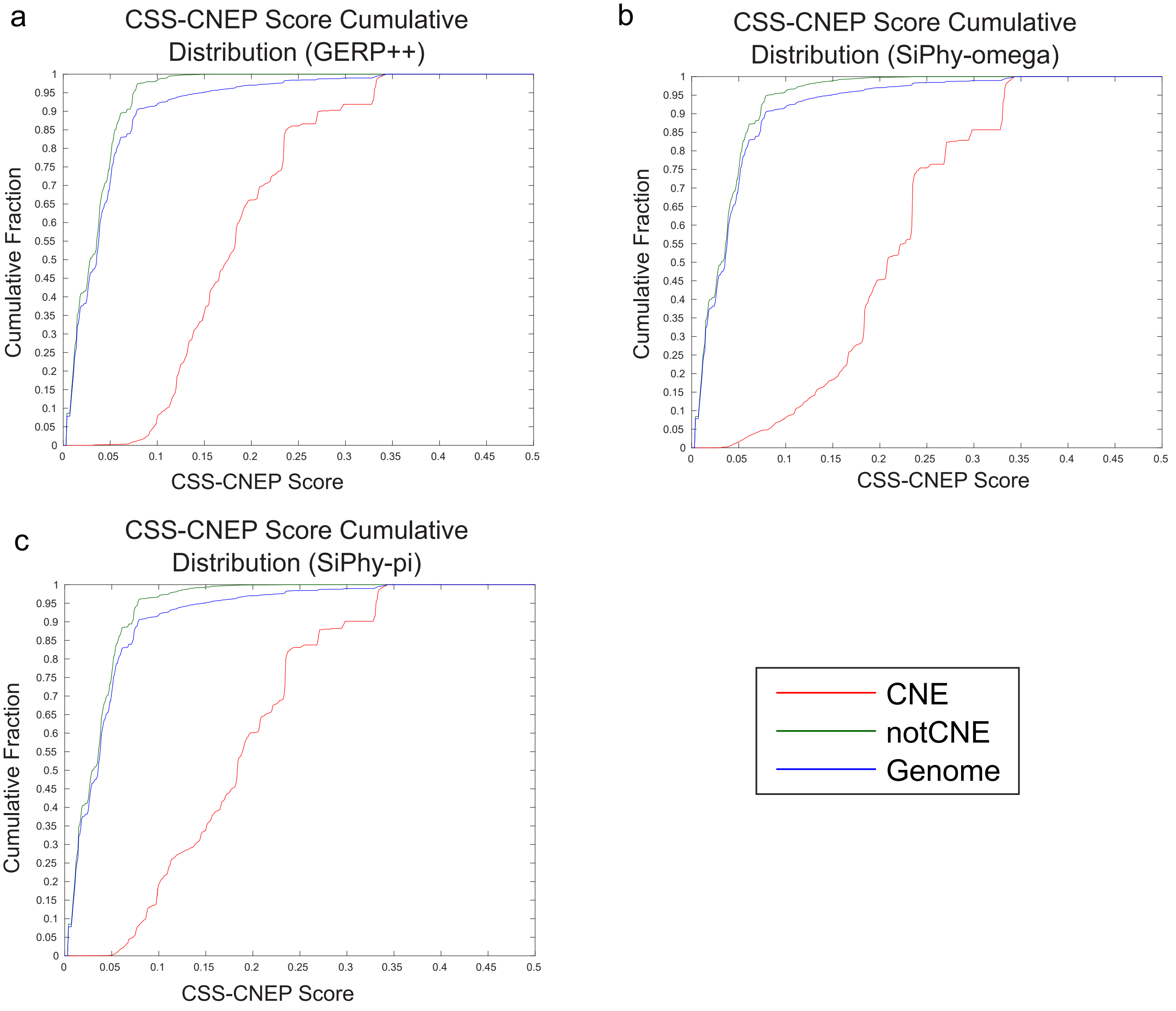

Supplementary Figure 25: Cumulative Distribution of CSS-CNEP Score. Similar plots as shown in **Fig. 7a** showing the cumulative distribution of the CSS-CNEP genomewide, in CNE, and notCNE bases, but shown here for **(a)** GERP++, **(b)** SiPhy-omega, and **(c)** SiPhy-pi constrained element sets. Source data are provided as a Source Data file.

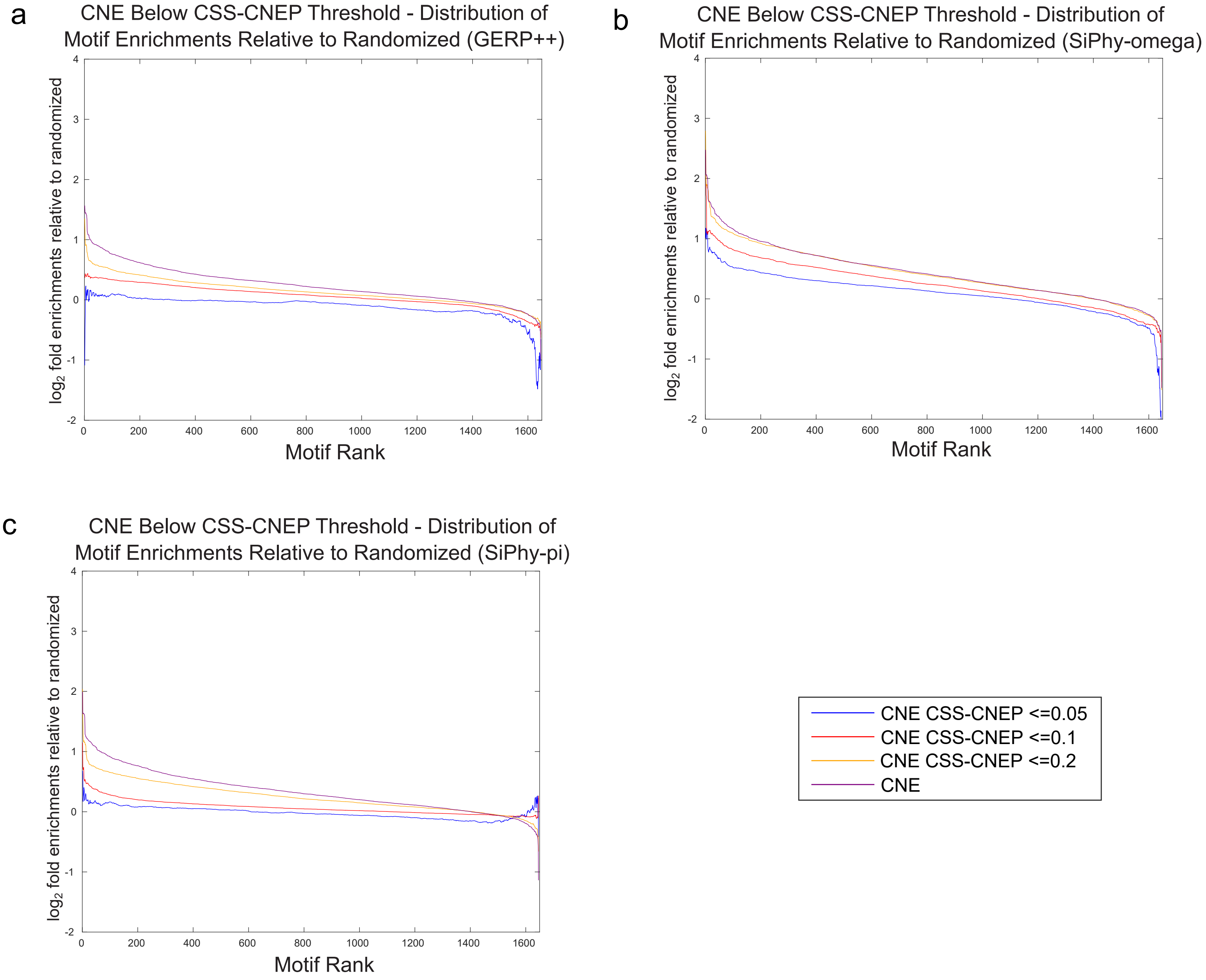

Supplementary Figure 26: CSS-CNEP and distribution of CNE motif enrichments. Similar plots as shown in **Fig. 7c** showing the difference of the distribution of motif enrichments relative to the distribution for a randomized set of the motifs for CNE bases and the subsets that had CSS-CNEP scores <=0.05, 0.10, and 0.20, but shown here for **(a)** GERP++, **(b)** SiPhy-omega, and **(c)** SiPhy-pi constrained element sets. Source data are provided as a Source Data file.

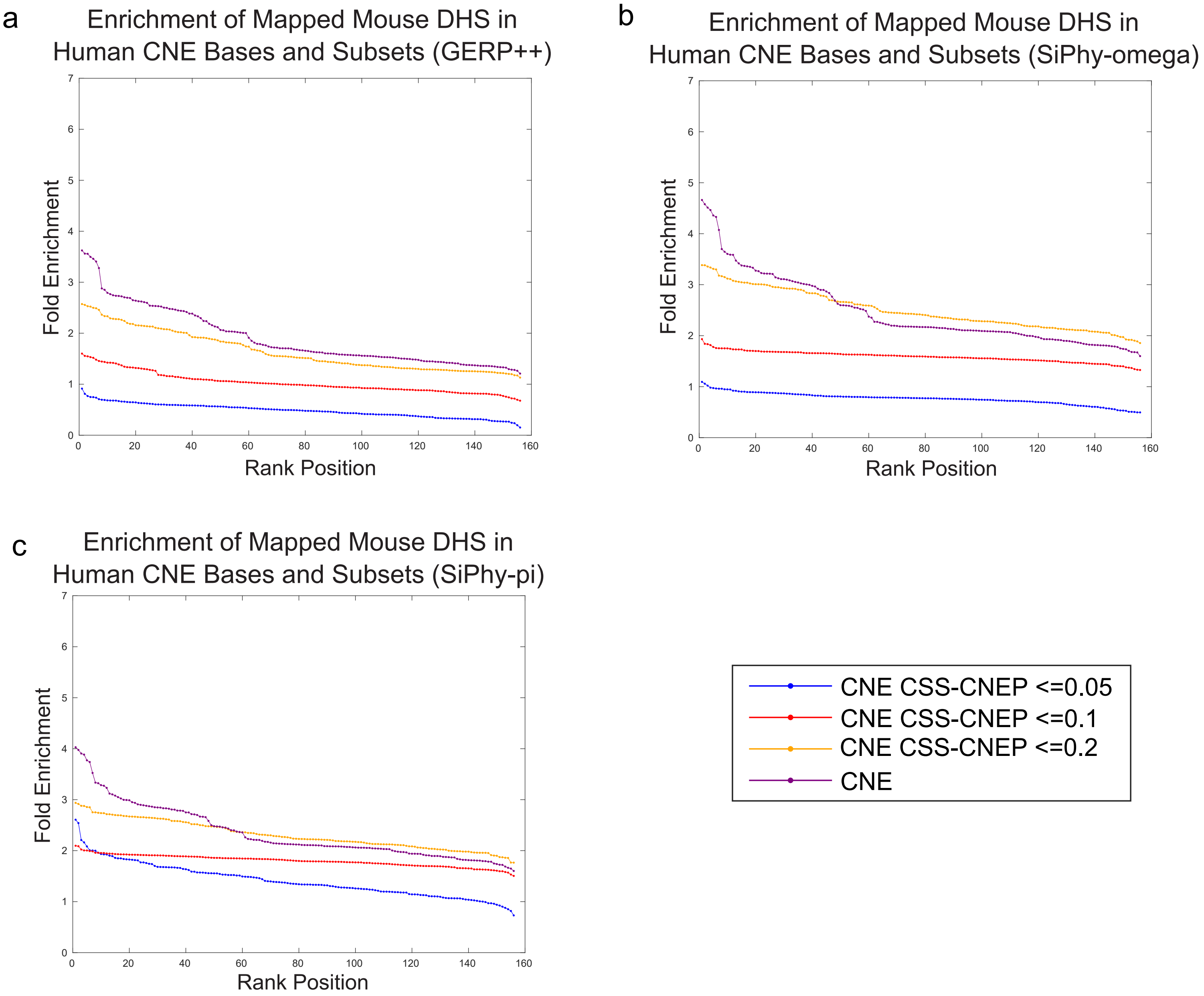

Supplementary Figure 27: CSS-CNEP and distribution of CNE mapped mouse DHS enrichments. Similar plots as shown in **Fig. 7d** showing enrichments of DNase I Hypersensitive Sites (DHS) from 156 mouse experiments mapped to human for CNE bases and the subsets that had CSS-CNEP scores <=0.05, 0.10, and 0.20, but shown here for **(a)** GERP++, **(b)** SiPhy-omega, and **(c)** SiPhy-pi constrained element sets. Source data are provided as a Source Data file.

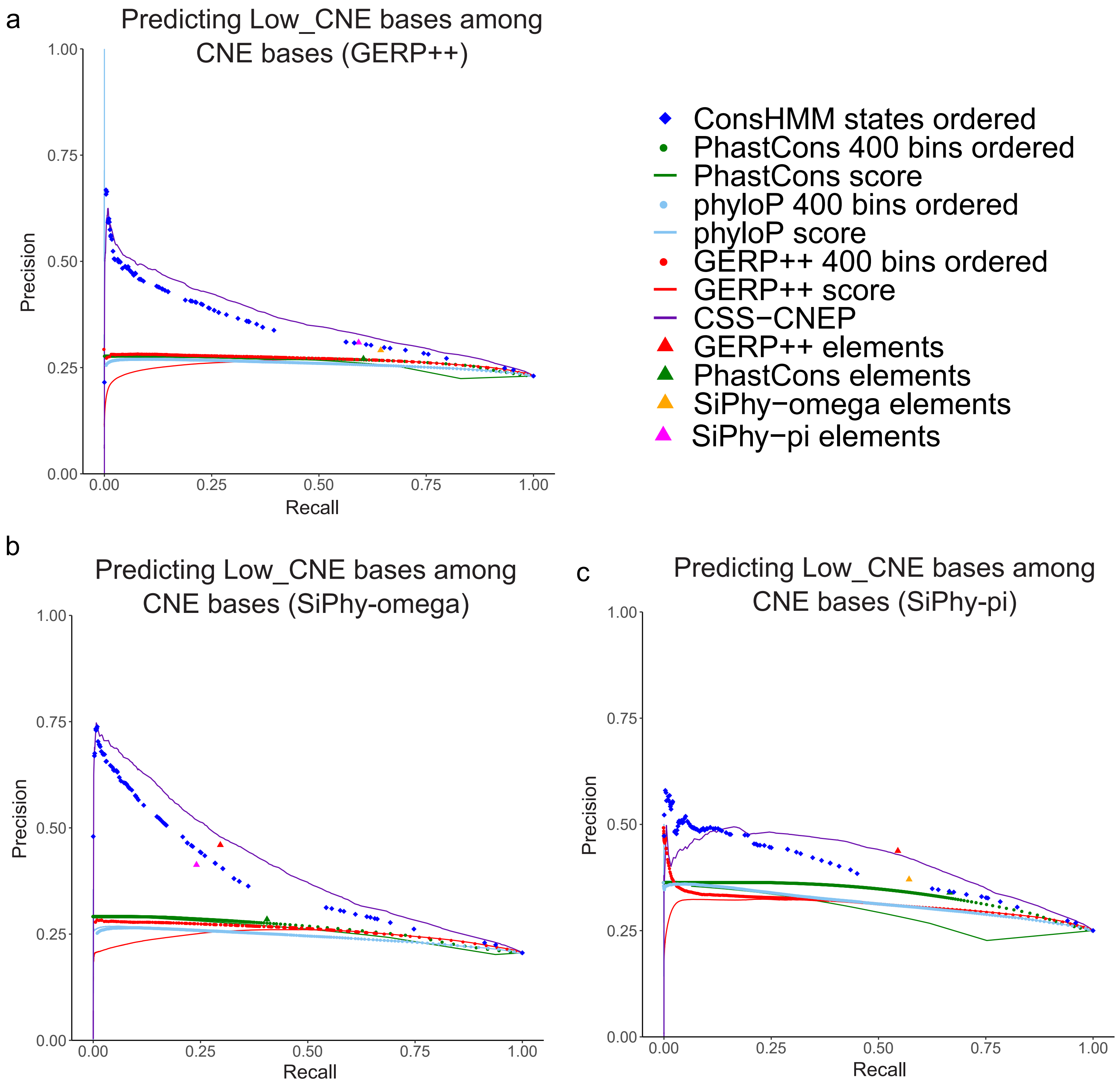

Supplementary Figure 28: Predictions of Low_CNE bases among CNE bases. Similar plots as shown in **Fig. 7b** showing the prediction of Low_CNE bases among CNE bases by the CSS-CNEP score and other comparative genomic annotations, but shown here for **(a)** GERP++, **(b)** SiPhy-omega, and **(c)** SiPhy-pi constrained element sets. Source data are provided as a Source Data file.

Method	Exons included in Negatives: PhastCons	Exons included in Negatives: GERP++	Exons included in Negatives: SiPhy-omega	Exons included in Negatives: SiPhy-pi		Exons excluded from Negatives and Positives: PhastCons	Exons excluded from Negatives and Positives: GERP++	Exons excluded from Negatives and Positives: SiPhy-omega	Exons excluded from Negatives and Positives: SiPhy-pi
CNEP	0.79	0.86	0.86	0.84		0.79	0.86	0.87	0.84
CNEP - matched	0.79	0.86	0.86	0.84		0.79	0.86	0.86	0.84
CNEP - five classifiers per element set	0.79	0.86	0.86	0.84		0.79	0.86	0.86	0.84
CNEP - single classifier per element set	0.76	0.83	0.83	0.80		0.76	0.83	0.83	0.80
CNEP - 2015 features only	0.75	0.82	0.82	0.81		0.75	0.82	0.83	0.81
CNEP - excluding ChromHMM features	0.78	0.86	0.86	0.83		0.78	0.86	0.86	0.83
CNEP - ChromHMM features only	0.70	0.76	0.77	0.76		0.70	0.76	0.77	0.76
All Features Count	0.62	0.64	0.68	0.69		0.62	0.64	0.68	0.69
All DNase-seq Count	0.67	0.71	0.74	0.74		0.67	0.71	0.75	0.75
Roadmap Epigenomics DNase-seq Count	0.67	0.71	0.74	0.73		0.67	0.72	0.74	0.74
Segway Encyclopedia - 'conservation-associated activity score'	0.57	0.60	0.64	0.66		0.58	0.61	0.66	0.67
FitCons2 - 'cell-type integrated score'	0.57	0.58	0.62	0.62		0.58	0.59	0.63	0.63
CNEP - on sample of 50,000 data points genomewide	0.81	0.84	0.87	0.84		0.81	0.84	0.87	0.85
DeepSea max data set - on sample of 50,000 data points genomewide	0.64	0.65	0.66	0.66		0.65	0.66	0.67	0.67
DeepSea average data set - on sample of 50,000 data points genomewid	0.52	0.53	0.53	0.54		0.53	0.53	0.55	0.54
CNEP - on sample of 1 million data points on chr10	0.81	0.84	0.86	0.84		0.81	0.84	0.86	0.84
Random Forest - on sample of 1 million data points on chr10	0.77	0.80	0.82	0.81		0.77	0.80	0.83	0.82

Supplementary Table 1: AUC performance at predicting CNE bases. The table reports the area under the ROC curves (AUC) of CNEP and other scores for predicting CNE bases. AUC values are shown for four constrained non-exonic element sets when bases overlapping exons are included in the negative set (left) and when they are excluded from both the positive and negative sets for the evaluations (right). The first row of the table provides the AUC values for the CNEP score. The second row of the table shows the AUC values for CNEP predictions based on training on only the constrained element set used in the evaluations instead of averaging predictions based on training on four different constrained elements sets. The next two rows show the predictive performance when CNEP uses five and one classifier per chromosome instead of ten. The next row shows the performance of CNEP with the feature subset used in the retrospective analysis, all of which were available by 2015. The following two rows report the performance of CNEP using all features except ChromHMM features and the performance of CNEP when using only the ChromHMM features. The next three rows report the performance of three baselines based on counting either all overlapping features, all overlapping DNase I features, or just overlapping Roadmap Epigenomics DNase I features. The following two rows report the performance of two existing scores, the Segway Encyclopedia 'conservation-associated activity score'[3] and FitCons2 'cell-type integrated scores'[4]. After the section break, three rows report the performance on a sample of 50,000 randomly sampled genomic positions. The first of those is the CNEP score followed by the maximum and average performance of DeepSea predictions for 919 chromatin features[5]. In the last section, two rows that report performance on a million randomly sampled positions on chr10. These rows report CNEP's performance using a logistic regression classifier and using a random forest classifier in place of a logistic regression classifier. Source data are provided as a Source Data file.

Base Set	Definition
CNE	bases in a constrained element that do not overlap a GENCODE exon
Low_CNE	bases in a constrained element that do not overlap a GENCODE exon and have a CNEP score below average (less than or equal to 0.0419)
High_CNE	bases in a constrained element that do not overlap a GENCODE exon and have a CNEP score above average (greater than 0.0419)
notCNE	bases not in a constrained element and do not overlap a GENCODE exon
Low_notCNE	bases not in a constrained element and do not overlap a GENCODE exon and have a CNEP score below average (less than or equal to 0.0419)
High_notCNE	bases not in a constrained element and do not overlap a GENCODE exon and have a CNEP score above average (greater than 0.0419)

Supplementary Table 2: Base set definition. This table provides the definitions of CNE, Low_CNE, High_CNE, notCNE, Low_notCNE, and High_notNCE bases for a given constrained element set.

Category Name	p-value	Corrected p-value	Fold
Pluripotent stem cell	4.70E-13	1.00E-11	3.1
Pancreas	2.90E-04	0.006	4.2
Neural	1.20E-03	0.026	2.2
Prostate	0.02	0.412	1.8

Supplementary Table 3: ChIP-Atlas cell type class enrichments. The table reports the most significant cell type class enrichments for the 209 ChIP-Atlas datasets that had a CNEP underestimation value greater than 0.02 restricting to those datasets that covered at least 200kb. The background consisted of the 19,604 datasets that covered at least 200kb. The columns are the name of the cell type class, uncorrected p-value, corrected p-value, and fold enrichment. P-values were computed by STEM[7] based on a one-sided test using the hypergeometric distribution. A Bonferonni correction was used to determine corrected p-values. Source data are provided as a Source Data file.

Supplementary References

1. Arneson, A. & Ernst, J. Systematic discovery of conservation states for single-nucleotide annotation of the human genome. *Commun. Biol.* **2**, 248 (2019).
2. Ernst, J. & Kellis, M. Large-scale imputation of epigenomic datasets for systematic annotation of diverse human tissues. *Nat. Biotechnol.* **33**, 364–376 (2015).
3. Libbrecht, M. W. *et al.* A unified encyclopedia of human functional DNA elements through fully automated annotation of 164 human cell types. *Genome Biol.* **20**, 180 (2019).
4. Gulko, B. & Siepel, A. An evolutionary framework for measuring epigenomic information and estimating cell-type specific fitness consequences. *Nat. Genet.* **51**, 335–342 (2019).
5. Zhou, J. & Troyanskaya, O. G. Predicting effects of noncoding variants with deep learning-based sequence model. *Nat. Methods* **12**, 931–934 (2015).
6. Kheradpour, P. & Kellis, M. Systematic discovery and characterization of regulatory motifs in ENCODE TF binding experiments. *Nucleic Acids Res.* **42**, 2976–2987 (2014).
7. Ernst, J. & Bar-Joseph, Z. STEM: a tool for the analysis of short time series gene expression data. *BMC Bioinformatics* **7**, 191 (2006).
