# Supplementary material for: Identification and characterization of constrained non-exonic bases lacking predictive epigenomic and transcription factor binding annotations
Source: Nat Commun. 2020 Dec 2;11:6168. doi: 10.1038/s41467-020-19962-9 (PMC7710766; doi:10.1038/s41467-020-19962-9)
Supplement: Supplementary file 3 — Description of Additional Supplementary Files [file 41467_2020_19962_MOESM3_ESM.pdf]

Description of Additional Supplementary Files

Supplementary Data 1: Input Features. The first sheet summarizes the sources of the epigenomics and TF binding data used as features to CNEP. Each feature corresponds to one file matching the URL up to the wild card (‘*’) except as indicated for the chromatin state and ReMap data. The last column indicates if the set of features were held out from training in the retrospective analysis. The remaining sheets list the individual features separately for data from the ENCODE portal, ChIP-Atlas, ChromHMM, Roadmap Epigenomics peaks calls, Roadmap Epigenomics digital genomics footprint calls, ENCODE peak calls not from the portal, ReMap 2015, and ReMap 2018. For ENCODE portal and ChIP-Atlas a feature is denoted by an accession, but metadata is also provided.

Supplementary Data 2: Observed and Expected average feature score for input features. This data reports the observed average CNEP score for all 63,741 binary features provided to CNEP in bases in which the feature is positive. The data also reports the expected average CNEP score for the feature, which is the genome-wide frequency of constrained non-exonic elements overlapping bases in which the feature is present, averaged over the four different constrained element sets considered. The last column reports the number of bases in the genome for which the feature was positive. The features are listed in decreasing order of the observed average CNEP score.

Supplementary Data 3: Motif enrichments. This data reports the $\log_2$ fold enrichment for 1,646 motifs for bases in Low_CNE and High_CNE for each of the four constrained element sets considered. Motifs are ordered based on the enrichment in Low_CNE bases for PhastCons.

Supplementary Data 4: TF Motif GO enrichments. For each constrained element set, there is a separate sheet with three tables reporting GO enrichments for TF subsets corresponding to three motif subsets. The subsets, in order, correspond to ‘High_CNE strongly preferred’, ‘High_CNE moderately preferred’, and ‘Low_CNE motif’ subsets. The columns are the ID of the GO category, GO category name, uncorrected p-value, corrected p-value, and fold enrichment. Only GO enrichments that had a corrected p-value <=0.10 are shown. P-values were computed by STEM based on a one-sided test using the hypergeometric distribution. The background set had 585 unique TFs, and the number of unique TFs in each set is provided in the data file. A Bonferonni correction was used to determine corrected p-values.

Supplementary Data 5: Mouse DHS enrichments for human Low_CNE bases. This data reports the fold enrichment of mouse DHS mapped to human in Low_CNE bases relative to a randomized version of the DHS. Included in the table are enrichments for the four constrained element sets considered for each of the 156 mouse DNase I hypersensitivity experiments.

Supplementary Data 6: Retrospective analysis of information in additional human datasets. This data reports separately for each dataset included in the ChIP-atlas, ENCODE portal, and ReMap 2018 compendia as well as for GENCODE exons present in v28 and not v19: (i) observed average CNEP score, (ii) expected average CNEP score, (iii) number of base pairs covered by the dataset, (iv) difference between the expected and observed average CNEP score. For these analyses the CNEP score was derived based on 10,836 features available as of 2015. Additionally, the antigen, cell type, and cell type class from the database provided metadata is also reported for ChIP-Atlas. The assay, biosample, and where applicable experiment target from the metadata are reported for the ENCODE portal data. For ReMap, datasets, information about the dataset is included in the dataset ID.
