# Supplementary material for: Identification and characterization of constrained non-exonic bases lacking predictive epigenomic and transcription factor binding annotations
Source: Nat Commun. 2020 Dec 2;11:6168. doi: 10.1038/s41467-020-19962-9 (PMC7710766; doi:10.1038/s41467-020-19962-9)
Supplement: Supplementary file 10 — Reporting Summary [file 41467_2020_19962_MOESM10_ESM.pdf]

nature research

Corresponding author(s): Jason Ernst

Last updated by author(s): Sep 9, 2020

Reporting Summary

Nature Research wishes to improve the reproducibility of the work that we publish. This form provides structure for consistency and transparency in reporting. For further information on Nature Research policies, see Authors & Referees and the Editorial Policy Checklist .

Statistics

For all statistical analyses, confirm that the following items are present in the figure legend, table legend, main text, or Methods section.

n/a	Confirmed	
x		The exact sample size (*n*) for each experimental group/condition, given as a discrete number and unit of measurement
x		A statement on whether measurements were taken from distinct samples or whether the same sample was measured repeatedly
	x	The statistical test(s) used AND whether they are one- or two-sided<br>*Only common tests should be described solely by name; describe more complex techniques in the Methods section.*
	x	A description of all covariates tested
	x	A description of any assumptions or corrections, such as tests of normality and adjustment for multiple comparisons
	x	A full description of the statistical parameters including central tendency (e.g. means) or other basic estimates (e.g. regression coefficient) AND variation (e.g. standard deviation) or associated estimates of uncertainty (e.g. confidence intervals)
	x	For null hypothesis testing, the test statistic (e.g. *F*, *t*, *r*) with confidence intervals, effect sizes, degrees of freedom and *P* value noted<br>*Give P values as exact values whenever suitable.*
x		For Bayesian analysis, information on the choice of priors and Markov chain Monte Carlo settings
x		For hierarchical and complex designs, identification of the appropriate level for tests and full reporting of outcomes
x		Estimates of effect sizes (e.g. Cohen's *d*, Pearson's *r*), indicating how they were calculated

Our web collection on statistics for biologists contains articles on many of the points above.

Software and code

Policy information about availability of computer code

Data collection: All data used in the manuscript is publicly available so no software was used to collect data.

Data analysis: The CNEP software (v1.0) is available at https://github.com/ernstlab/CNEP. Liblinear v.2.1, which is used by CNEP is available https://www.csie.ntu.edu.tw/~cjlin/liblinear/. The STEM software (v1.3.11) used for GO enrichment analysis is available at http://sb.cs.cmu.edu/stem/. The ChromHMM software (v1.13) used for computing state enrichments is available from https://ernstlab.biolchem.ucla.edu/ChromHMM/. Scikit-learn (v.0.19) used for the Random Forest classification is available from https://scikit-learn.org/. BEDTools (v.2.17) used for shuffling bed files is available from https://github.com/arq5x/bedtools2. DeepSea was run through its web interface available at http://deepsea.princeton.edu/job/analysis/create/.

For manuscripts utilizing custom algorithms or software that are central to the research but not yet described in published literature, software must be made available to editors/reviewers. We strongly encourage code deposition in a community repository (e.g. GitHub). See the Nature Research guidelines for submitting code & software for further information.

Data

Policy information about availability of data

All manuscripts must include a data availability statement. This statement should provide the following information, where applicable:

- Accession codes, unique identifiers, or web links for publicly available datasets
- A list of figures that have associated raw data
- A description of any restrictions on data availability

Data availability

The CNEP and the CSS-CNEP scores are available from https://github.com/ernstlab/CNEP. All input data used to generate the scores are publicly available. Identifiers for the features are provided in the Supplementary Data 1. SiPhy-pi and SiPhy-omega constrained elements were from https://www.broadinstitute.org/mammals-models/29-mammals-project-supplementary-info. GERP++ constrained elements and scores were from http://mendel.stanford.edu/SidowLab/downloads/gerp/. PhastCons constrained elements and scores, PhyloP scores, and RepeatMasker annotations were from the UCSC genome browser (https://

genome.ucsc.edu/). The main exon annotations were from ftp://ftp.sanger.ac.uk/pub/gencode/Gencode_human/release_19/gencode.v19.annotation.gtf.gz and exon annotations from the retrospective analyses were from ftp://ftp.ebi.ac.uk/pub/databases/gencode/Gencode_human/release_28/GRCh37_mapping/gencode.v28lift37.annotation.gtf. ChIP-Atlas data were from http://dbarchive.biosciencedbc.jp/kyushu-u/hg19/eachData/bed05. ReMap data were from http://remap.univ-amu.fr/. Roadmap Epigenomics data were from http://compbio.mit.edu. ENCODE consortium data were from http://hgdownload.cse.ucsc.edu/goldenPath/hg19/encodeDCC and http://www.broadinstitute.org/~anshul/projects/encode/rawdata/peaks_histone/mar2012/narrow/combrep_and_ppr/ and for the ENCODE portal portion from https://www.encodeproject.org/. 1000 Genomes Phase 3 data is available at ftp://ftp.1000genomes.ebi.ac.uk/vol1/ftp/release/20130502/. Regulatory motif data is from http://compbio.mit.edu/encode-motifs/matches-with-controls.txt.gz. Mouse ENCODE data were from http://hgdownload.soe.ucsc.edu/goldenPath/mm9/encodeDCC/wgEncodeUwDnase/ and http://hgdownload.soe.ucsc.edu/goldenPath/mm9/encodeDCC/wgEncodeUwDgf/. Mutation rate estimates were from http://mutation.sph.umich.edu/hg19/. B-values were from http://krishna.gs.washington.edu/download/CADD/v1.4/GRCh37/annotationsGRCh37.tar.gz Conservation state annotations and bases prioritized by various variant prioritization scores were from https://github.com/ernstlab/ConsHMM/. Segway Encyclopedia and FitCons2 scores were from https://noble.gs.washington.edu/proj/encyclopedia/caas.bed.gz and http://compgen.cshl.edu/fitCons2/hg19/H1/E999-sco.bw, respectively. Source data for figures are available in the Source Data file.

Field-specific reporting

Please select the one below that is the best fit for your research. If you are not sure, read the appropriate sections before making your selection.

- [x] Life sciences
- [ ] Behavioural & social sciences
- [ ] Ecological, evolutionary & environmental sciences

For a reference copy of the document with all sections, see nature.com/documents/nr-reporting-summary-flat.pdf

Life sciences study design

All studies must disclose on these points even when the disclosure is negative.

Sample size	A sample size was not needed to be determined for this study.
Data exclusions	No data was excluded.
Replication	We observed high correlation of predictions when training on different constrained element sets individually . We also observed high correlations for predictions when generating two sets of predictions based on splitting the ensemble of ten classifiers per element set into two-halves.
Randomization	This is not relevant since there was no experimental groups in this study.
Blinding	This is not relevant since there was no group allocation as part of this study.

Reporting for specific materials, systems and methods

We require information from authors about some types of materials, experimental systems and methods used in many studies. Here, indicate whether each material, system or method listed is relevant to your study. If you are not sure if a list item applies to your research, read the appropriate section before selecting a response.

Materials & experimental systems

n/a	Involved in the study	
[x]	[ ]	Antibodies
[x]	[ ]	Eukaryotic cell lines
[x]	[ ]	Palaeontology
[x]	[ ]	Animals and other organisms
[x]	[ ]	Human research participants
[x]	[ ]	Clinical data

Methods

n/a	Involved in the study	
[x]	[ ]	ChIP-seq
[x]	[ ]	Flow cytometry
[x]	[ ]	MRI-based neuroimaging
